# Comparison of the Effect of Two Kinds of Iranian Honey and Diphenhydramine on Nocturnal Cough and the Sleep Quality in Coughing Children and Their Parents

**DOI:** 10.1371/journal.pone.0170277

**Published:** 2017-01-19

**Authors:** Parviz Ayazi, Abolfazl Mahyar, Mahdieh Yousef-Zanjani, Abbas Allami, Neda Esmailzadehha, Taraneh Beyhaghi

**Affiliations:** 1 Children Growth Research Center, Qazvin University of Medical Sciences, Qazvin, Iran; 2 Department of Pediatrics, Qazvin University of Medical Sciences, Qazvin, Iran; 3 Department of Infectious Diseases, Qazvin University of Medical Sciences, Qazvin, Iran; 4 Metabolic Diseases Research Center, Qazvin University of Medical Sciences, Qazvin, Iran; Charité - Universitätsmedizin Berlin, GERMANY

## Abstract

Coughing in a child induced by upper respiratory tract infections (URTIs) can be a problem, both for the child and its parents. Current studies show a lack of proven efficacy for over-the counter (OTC) medications, but promising data support the use of honey for children. The aim of this study was to compare the effects of two kinds of Iranian honey with diphenhydramine (DPH) on nocturnal pediatric coughs and the sleep quality of children and their parents. This was a clinical trial (registered in IRCT; No.: 28.20.7932, 15 October 2013). The study consisted of 87 patients. All the parents completed a standard previously validated questionnaire. The children were randomly assigned to one of three treatment groups: Group 1, Honey type 1 (Kimia Company, Iran) (*n* = 42), Group 2, Honey type 2 (Shahde-Golha, Iran) (*n* = 25), and Group 3, DPH (*n* = 20). Each group received double doses of the respective treatments on two successive nights. A second survey was then administered via a telephone interview in which the parents were asked the same questions. The mean scores for all aspects of coughs were significantly decreased in each group before and after the treatment. All three treatments improved the cough and sleep scores. Honey type 1 was superior to DPH in improving all aspects of coughs, except the frequency, and Honey type 2 was more effective than DPH in improving all aspects of coughs, except the sleep quality of the child. There was no significant difference between Honey type 1 and 2 in any aspects of cough relief in the present study. The results suggest that honey may provide better cough relief than DPH in children and improve the sleep quality of children and their parents.

## Introduction

A cough is a normal protective mechanism of the respiratory system to eliminate excessive secretions and foreign bodies. The causes of cough can be bacterial or viral infections and/or the presence of an irritant or allergen in the respiratory tract [1]. Upper respiratory tract infections (URTIs) are prevalent among the pediatric age group and usually occur 6–8 times per year. URTIs are one of the most common causes of coughing among children [2]. Viruses are generally the cause of URTIs, with bacteria the causative agent in less than 10% of cases [3]. Rhinorrhea, malaise, a low-grade fever, sneezing, and coughing are the typical signs and symptoms of viral disease. Most of these symptoms appear during the first 3 days and disappear within a week. However, the cough may persist for weeks [2,4].

Coughing, especially during the night, can be particularly troubling to children and their parents, as it often results in discomfort to the child and loss of sleep for both the child and its parents. As a result, children may miss daycare or school, and parents may have to take time off work. The desire of parents for an effective and speedy intervention tends to lead to the use of over-the- counter (OTC) cough medications [4]. However, these medications are potentially dangerous, with many reported adverse events caused by inadvertent overdoses when parents administer more than the recommended frequency or dose to the child [5].

There is no agreement on the treatment of URTIs and related coughing. There is also no consensus on the cough-relief ability of diphenhydramine (DPH) [3]. The lack of proven efficacy for OTC medications, together with the fact that they are not approved by professional organizations, such as the American Academy of Pediatrics [6] and the Food and Drug Administration (FDA) [7], has led many researchers to investigate the efficacy of other remedies for URTI-related coughs [4,5,8–11].

Honey has long been recommended by traditional medicine for the treatment of URTI-related coughs [10–12]. According to the World Health Organization (WHO), honey is a treatment choice for coughs and URTI symptoms. Except in infancy, honey has been shown to be a useful, inexpensive, readily available, and safe substance for children [13]. Honey has antioxidant ingredients, which result in antimicrobial and anti-inflammatory effects [14,15]. The aim of this study was to compare the effects of two kinds of Iranian honey with diphenhydramine (DPH) on nocturnal pediatric coughs due to viral URTIs and the sleep quality of children and their parents.

## Materials and Methods

This was a clinical trial study, which began in September 2013 and ended in October 2014. The participants were 1–12 years old children who attended the pediatrics outpatient clinic of the children’s hospital affiliated to Qazvin University of Medical Sciences in Qazvin, Iran. The ethics committee of Qazvin University of Medical Sciences approved the study protocol (No.: 28.20.7932, 15 October 2013), and it was performed in accordance with the ethical standards laid down in the 1964 Declaration of Helsinki and its later amendments. The parents were given an information sheet about the study protocol before they entered the trial and informed that they could withdraw from the study at any point. All the parents provided written informed consent on behalf of their children.

**Fig 1:**
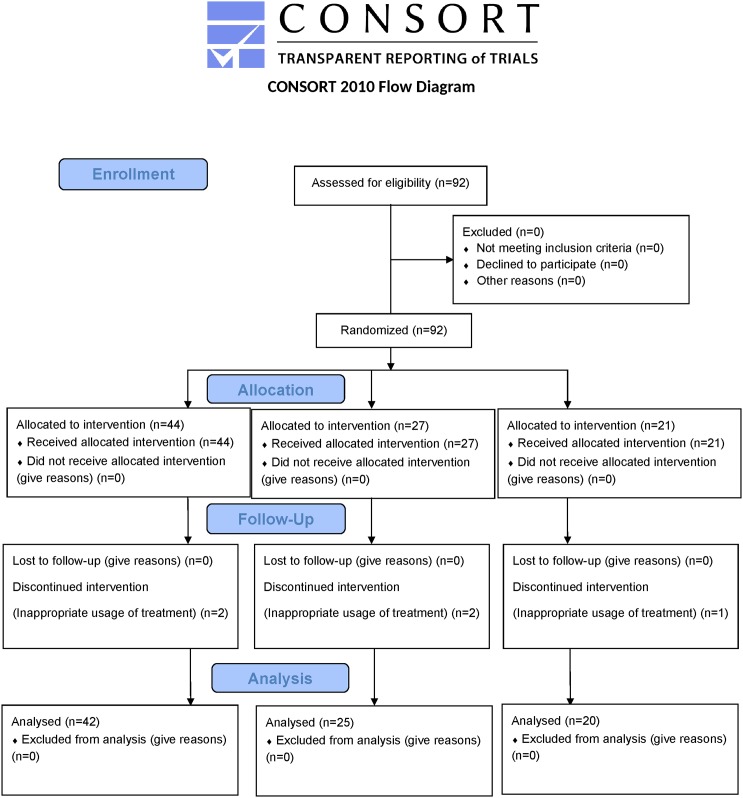
CONSORT flowchart of the study.

This trial had two main parts. In the first part, patients were informed about the research project via a banner, which was placed in the pediatrics outpatient clinic from 23 September 2013 to 23 December 2013. In the second part, the patients’ recruitment began in 23 December 2013, and the follow-up period ended in 23 October 2014. When the study protocol was approved by the aforementioned ethics committee, the trial registry was mandatory, but the registration time was optional in Qazvin University of Medical Sciences. Therefore, this clinical trial was registered with the IRCT retrospectively after the patients’ recruitment. All ongoing and related trials on this drug/intervention have been registered. There was not any deviation from the approved study protocol. The Consolidated Standards of Reporting Trials (CONSORT) flowchart of the study is shown in Fig 1.

The estimated sample size necessary to detect a 1-point difference between any treatment groups with 80% power, α = 0.05, based on a 2-sided and 1-point difference for the primary outcome has been used previously (two well-known and similar clinical trials) [10,11]. Only 11 patients are needed for each group in order to prove the difference within groups (Paired T test), but 20–30 patients are needed in each group to be able to show minimum difference of 1 between three groups (multiple comparison test).

The inclusion criteria were the presence of a viral URTI-induced cough in a previously healthy subject with no past medical history, which had to have lasted up to 7 days, with or without rhinorrhea. The manifestations of viral URTIs included congestion, fever (an oral temperature of less than 39°C), pharyngitis, malaise, or headaches. The exclusion criteria were signs and symptoms compatible with pneumonia, laryngotracheobronchitis, sinusitis, asthma, allergic rhinitis, frequent hospitalization, recent administration of DPH to children, and use of medications that affected the sleep of the parents (e.g. benzodiazepines and antihistamines). Patients were also excluded if they had used any cough or cold medication or honey on the night before entering the study. Patients were not excluded when analgesic medications, such as acetaminophen or ibuprofen, were administered on either night of the study. Patients were selected by a pediatrician who was completely aware of the study design.

**Fig 2:**
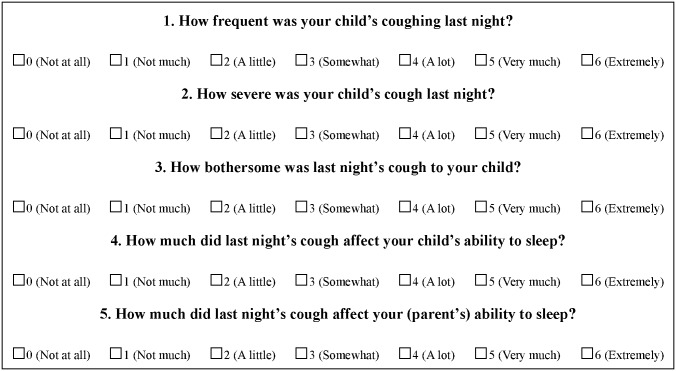
Nocturnal cough and sleep difficulty assessment questionnaire.

After informing the participants, nocturnal coughing and sleep difficulty were assessed using a Persian version of a standard previously validated questionnaire [10,16]. This was a 5-item questionnaire, which assessed the child’s cough and sleep difficulty on a 7-point Likert scale (Fig 2). All the parents completed the questionnaire, which asked them about their subjective assessments of the child’s coughing and the child’s and their own sleep quality the previous night. The minimum symptom severity criterion for entering the trial was a severity score of three on a minimum of two questions related to nocturnal cough frequency, effect on the child’s sleep, and effect on parental sleep the previous night.

After stratification for age (1–6 years and 6–12 years), eligible patients were selected by the aforementioned pediatrician, and their parents were given one colored card to prevent parents from knowing which group their child would belong. Then the parents hand the colored card to the project presenter. Yellow, pink, and blue colored cards represented Honey type 1, Honey type 2, and DPH, respectively. The presenter then gave the related treatment container to the parents. If a parent asked about the treatment type, the presenter revealed the identity of the treatment. The study was not blinded due to the appearance and taste of the treatment types.

The treatment type was identical for up to two weeks. The number of participants in each group was dependent on the number of patients referred to the clinic. The first treatment was Honey type 1, followed by Honey type 2, and DPH. The allocation was continued every two weeks until at least 20 patients were entered in each group.

Two groups were given two different types of honey in a 10 mL opaque plastic container. Group 1 was given Kimia honey (No. 4916, Kimia, Ardabil, Iran), and Group 2 was given Shahde-Golha honey (No. 73294, Shahde-Golha, Khorasan Razavi, Iran). Ardabil and Khorasan Razavi are located in Northwest and Northeast of Iran, respectively. The third group (control group) received an oral solution of DPH (Ramopharmin, Tehran, Iran) in a 10 mL opaque plastic container. The honey and DPH were stored at room temperature of about 22–26°C. The participants received their treatments 30 min before going to bed on two consecutive nights (two doses).

The younger children (aged 1–6 years) received a DPH dose of 6.25 mg/dose (2.5 mL), and the older children (6–12 years) received a dose of 12.5 mg/dose (5 mL). In the honey groups, the volume of honey dispensed was equivalent to the age-driven volume for DPH. Subjects who had consumed an inappropriate dose of the treatments for any reason were excluded from the analysis. The treatment condition was determined based on the parents' reports. Receiving the treatment for only one night or less than the prescribed volume was considered as inappropriate dose. After two nights of treatment, the parents completed the nocturnal cough and sleep difficulty questionnaire again via a telephone interview. The same parent answered the questions about symptom severity on both nights. No physical examination was performed the second time, unless it was made necessary due to illness progression. The main outcome measures were cough frequency, cough severity, bothersome nature of the cough, and the children’s and parents’ sleep quality.

Categorical variables were presented as numbers and relative frequencies (percentages) and differences between the groups were evaluated by Chi-square test. Continuous variables were presented as the mean plus or minus standard deviation (SD). The effects of the treatments on nocturnal coughing and sleep difficulty scores in each group were assessed using a paired T-test. The mean difference in the scores of nocturnal coughing and sleep difficulty before and after the treatment was calculated. The nocturnal cough and sleep difficulty scores were compared between groups using a multivariate analysis of variance. Age was considered a covariate. Post-hoc tests were performed using the LSD method for pairwise comparisons. All reported P-values were based on two-sided hypotheses and values less than 0.05 were considered statistically significant. All the analyses were performed using SPSS software, version 19 (SPSS, Inc., Chicago, IL, USA).

## Results

In total, 92 children were enrolled in the study and the mean age was 3.5±1.6 years. Of these 92 patients, 87 (94.6%) completed the two-night study and the final questionnaire, and none of these patients were excluded from the analysis. The other five (5.4%) patients (two in Group 1, two in Group 2, and one in Group 3) were excluded from the analysis due to inappropriate usage of the treatment (Fig 1). The number of patients lost to follow up was not significantly different between the groups (P>0.050).

The mean age of the patients who completed the study was 3.5±1.6 years, and the age difference between the groups was not statistically significant (P = 0.124). Of the patients, 46 (52.9%) were females. The mean duration of the URTIs before enrollment was 3.5±2.1 days. Treatment groups were similar with regards to sex and disease duration (P = 0.373 and P = 0.357, respectively). The comparison of the age-adjusted baseline cough characteristics of the children and sleep quality of children and their parents are shown in Table 1. The frequency of coughing was not significantly different between the three groups. The bothersome nature of the cough and sleep quality of the children were not statistically different between the honey groups.

**Table 1:** Comparison of the age-adjusted baseline scores of the three groups.

	Treatment group				Mean Difference		
Item	Honey 1(G1)(n = 42)	Honey 2(G2)(n = 25)	Diphenhydramine (G3)(n = 20)	P ^a^	G1 vs.G2 ^b^	G1 vs. G3	G2 vs. G3
Cough frequency	3.4 ± 0.9	3.7 ± 0.8	3.5 ± 1.2	0.321	-0.4	-0.1	0.3
Cough severity	3.7 ± 0.8	4.2 ± 0.7	3.7 ± 0.9	0.020	-0.5^c^	0.0	0.5^c^
Bothersome nature of cough	3.6 ± 0.9	3.6 ± 0.8	2.9 ± 0.9	0.010	-0.1	0.6^c^	0.7^c^
Sleep quality in child	3.1 ± 1.0	3.4 ± 1.3	2.5 ± 1.1	0.021	-0.3	0.6^c^	0.9^c^
Sleep quality in parents	3.7 ± 0.8	4.2 ± 0.7	2.7 ± 0.9	<0.001	-0.6^c^	0.9^d^	1.5^d^
Combined	17.5 ± 3.1	19.1 ± 3.9	15.0 ± 4.4	0.002	-1.9^c^	2.1^c^	4.0^d^

Data are presented as mean±SD.

^a^ Analysis of variance;

^b^ Post hoc multiple comparisons by LSD.

^c^ P< 0.050;

^d^ P< 0.001.

Scores of all cough related aspects decreased after interventions in the groups. (Table 2).

**Table 2:** Comparison of the scores before and after the intervention in the three groups.

Variables	Before	After	P ^a^
Cough frequency			
Honey 1	3.4 ± 0.9	1.9 ± 1.2	<0.001
Honey 2	3.7 ± 0.8	1.8 ± 1.5	<0.001
Diphenhydramine	3.4 ± 1.2	2.6 ± 1.6	0.002
Cough severity			
Honey 1	3.7 ± 0.8	2.0 ± 1.2	<0.001
Honey 2	4.2 ± 0.7	2.0 ± 1.8	<0.001
Diphenhydramine	3.6 ± 0.9	2.9 ± 1.2	0.002
Bothersome nature of cough			
Honey 1	3.6 ± 0.9	1.9 ± 1.2	<0.001
Honey 2	3.5 ± 0.8	1.5 ± 1.8	<0.001
Diphenhydramine	2.8 ± 0.9	2.2 ± 1.1	0.004
Sleep quality in child			
Honey 1	3.1 ± 1.0	1.0 ± 1.2	<0.001
Honey 2	3.4 ± 1.3	1.6 ± 2.1	0.001
Diphenhydramine	2.4 ± 1.1	1.5 ± 1.0	<0.001
Sleep quality in parents			
Honey 1	3.7 ± 0.8	1.5 ± 1.2	<0.001
Honey 2	4.2 ± 0.7	1.9 ± 1.4	<0.001
Diphenhydramine	2.6 ± 0.9	1.6 ± 0.9	<0.001
Combined			
Honey 1	17.5 ± 3.1	8.4 ± 5.7	<0.001
Honey 2	19.1 ± 3.9	8.9 ± 8.5	<0.001
Diphenhydramine	15.0 ± 4.4	10.9 ± 5.3	0.001

Data are presented as mean ± SD.

^a^ Paired t-test.

The comparison of the age-adjusted mean difference in the pre- and post-treatment scores of the groups is shown in Table 3. The mean differences between cough related aspects were significantly different between the three groups except for cough frequency and sleep quality in the children. Post hoc analysis showed that these differences are related to the differences of honey groups and DPH. Univariate analysis showed that mean differences in the scores of all cough related aspects before and after the intervention were not associated with duration of the URTIs before enrollment (P = 0.355, 0.544, 0.309, 0.339, and 0.353 for five cough related aspects, respectively). None of the participants reported treatment-related side effects during or after the study.

**Table 3:** Comparison of age-adjusted mean differences in the scores before and after the intervention in the three groups.

	Treatment group				Mean Difference		
Item	Honey 1 (G1) (n = 42)	Honey 2 (G2) (n = 25)	Diphenhydramine (G3) (n = 20)	P ^a^	G1 vs. G2 ^b^	G1 vs. G3	G2 vs. G3
Cough frequency	1.4±1.3	1.9±1.9	0.9±1.0	0.071	-0.5	0.5	1.0^c^
Cough severity	1.7±1.2	2.2±1.7	0.7±0.9	0.002	-0.5	1.0^c^	1.5^d^
Bothersome nature of cough	1.7±1.2	2.0±1.9	0.6±0.9	0.006	-0.4	1.0^c^	1.4^c^
Sleep quality in child	2.2±1.6	1.8±2.3	0.9±1.0	0.057	0.3	1.2^c^	0.8
Sleep quality in parents	2.1±1.3	2.2±1.4	1.0±1.0	0.003	-0.1	1.1^c^	1.2^c^
Combined	9.1±6.1	10.2±9.2	4.2±4.5	0.011	-1.2	4.8^c^	6.0^c^

Data are presented as mean±SD.

^a^ Analysis of variance;

^b^ Post hoc multiple comparisons by LSD.

^c^ P< 0.050;

^d^ P< 0.001.

## Discussion

Despite the common occurrence of URTIs and coughs, there are no accepted therapies for the latter [10], and most nonantibiotic treatments are probably not effective for coughs [17]. Studies have shown that dextromethorphan and DPH do not relieve the nighttime symptoms of URTIs [18]. Furthermore, these OTC medications have side effects, including somnolence, restlessness, overdoses, and unexpected deaths [5,19–23]. All the aforementioned data have led to the use of other non-OTC drugs or substances, including honey, for the treatment of URTI-related coughs.

In the present study, all three treatments improved both the cough and sleep scores. Honey type 1 was significantly superior to DPH in improving all cough-related aspects, except the cough frequency, and Honey type 2 was more effective than DPH in improving all cough-related symptoms, except the sleep quality of the child. In a study by Shadkam et al. in Iran, honey provided greater relief from URTI-induced coughs than dextromethorphan and DPH [16]. Although previous studies reported medication-related side effects, such as hyperactivity, nervousness, and gastrointestinal symptoms [4, 10], there were no reported side effects in the present study.

In a systematic review in 2014, Oduwole et al. evaluated the effectiveness of honey for acute coughs in children [24]. Based on only two eligible randomized controlled trial, the authors concluded that honey may be superior to “no treatment” or DPH in the clinical improvement of coughs. Finally, Oduwole et al. suggests that more RCTs on the use of honey in the treatment of cough in children are needed [24]. In the present study honey was effective in relieving most aspects of cough-related symptoms in children and their parents.

Honey has been shown to be a therapeutic option for children with acute coughs due to URTIs [25]. Honey is a sweet and viscous nutrient, which is composed of different substances [26], including free amino acids, trace elements, vitamins, flavonoids, and carbohydrates [27–29]. Various compounds contained in honey confer antioxidant properties [15,30]. It has also anti-inflammatory, antimicrobial, and antiviral actions [14,15,31]. Honey consumption induces reflexive saliva and mucus secretion in the airways, thereby relieving coughs, especially dry coughs [4,32]. Another possible explanation for the effects of honey on cough has been suggested by Eccles. There is a close anatomic relationship between the nerve fibers responsible for initiating cough and those responsible for tasting sweetness. Therefore sweet substances e.g. honey may have an antitussive effect via an interaction between these fibers [32]. The quantity of antioxidants varies in different types of honey due to their floral source and environmental factors [32]. However, in the present study, we found no significant difference between Honey type 1 and type 2 with regard to various aspects of cough relief.

As immunity against *Clostridium botulinum* is poor in infants, the use of honey is restricted [33]. Improvements in cough scores may partly be attributed to the natural history of URTIs, which generally improve with time and supportive care [10]. In the present study, disease duration was part of the inclusion criteria and in the final analysis the treatment groups were similar in terms of disease duration. On the other hand, in univariate analysis the mean differences in the scores of all cough related aspects were not associated with duration of the URTIs before enrollment. Therefore, the significant difference in relieving cough-related symptoms between honey and DPH groups indicates the therapeutic effects of honey on pediatric cough.

The use of two types of honey was strength of the present study since quantity of antioxidants varies in different types of honey due to their floral source and environmental factors. Another strength was the use of two successive doses of each type of honey since the intervention period was short in previous studies. This study has some limitations, including the difference in basal scores, which we controlled for in the analysis process, and the mean difference was compared between groups. In addition, the study was not double-blinded due to the appearance and taste of the treatment types. Considering the possibility that the honey quality may change and its effects may decrease after opening the sealed container, the allocation method was done as mentioned in the manuscript and random allocation was not possible that might be a source of bias.

In conclusion, the present study suggests that honey, may be superior to DPH in relieving cough symptoms in children and improving the sleep quality of children and therefore that of their parents. The results of the present study support the WHO’s recommendations about the use of honey as a potential treatment for coughs. More double-blind controlled trial studies, with larger sample sizes and longer durations of treatment, are necessary to investigate the therapeutic effect of honey on all aspects of cough relief in children.

## Supporting Information

S1 File CONSORT checklist of the study. (DOC)

S2 File Methods of proposal in Persian language. (DOC)

S3 File Methods of proposal in English language. (DOCX)

S4 File Study data in csv format. (CSV)

S5 File Vriable description of the dataset. (PDF)

S6 File Statistical analysis output of the study. (PDF)
