# Supplementary material for: Comparison of the Effect of Two Kinds of Iranian Honey and Diphenhydramine on Nocturnal Cough and the Sleep Quality in Coughing Children and Their Parents
Source: PLoS One. 2017 Jan 19;12(1):e0170277. doi: 10.1371/journal.pone.0170277 (PMC5245888; doi:10.1371/journal.pone.0170277)
Supplement: S2 File — (DOC) [file pone.0170277.s002.doc]

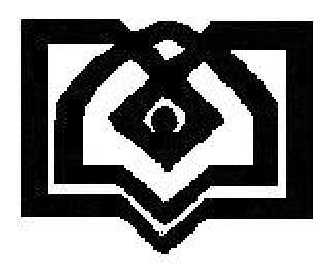
*بسمه تعالی*

# *دانشگاه علوم پزشكی و خدمات بهداشتی - درمانی قزوین*

*معاونت پژوهشی*

*فرم پیش نویس طرح پژوهشی*

عنوان طرح:

مقایسه اثر دو نوع عسل ایرانی و دیفن هیدرامین بر سرفه شبانه كوكان و كیفیت خواب شبانه این كودكان و والدین آنها

نام ونام خانوادگی مجریان:

دكتر پرویز ایازی، دكتر ابوالفضل مهیار، مهدیه یوسف زنجانی فرد

دانشکده/مرکز تحقیقاتی:

دانشكده پزشكی/ واحد حمایت از توسعه تحقیقات بالینی قدس

نوع مطالعه:

🗆کیفی ☑کارآزمایی بالینی 🗆اپیدمیولوژیک تحلیلی(مورد شاهدی،هم گروهی)

🗆تولیدی 🗆پایه (تجربی) 🗆اپیدمیولوژیک توصیفی 🗆مبتنی براطلاعات بیمارستانی

نحوه مشارکت:

☑ بدون مشارکت 🗆 چندمرکزی داخلی 🗆چند مرکزی خارجی

درصورت مشارکت نوع ونحوه همکاری وتامین اعتبار راذکر نمایید؟

تاریخ پیشنهاد: / /13 تاریخ شروع: همزمان با تاریخ تصویب

تاریخ تصویب: / / تاریخ خاتمه: 18 ماه پس از تاریخ تصویب مدت اجرا: 18 ماه

هزینه ها:

- هزینه پرسنلی: 8000000 ریال - هزینه وسایل: 1450000 ریال

- هزینه آزمایش ها: _ - هزینه کل: 10000000 ریال

آیا طرح درراستای اولویت های پژوهشی دانشگاه است؟

🗆بلی ( اولویت شماره....... صفحه........ )

🗆 خیر

آیا طرح نیاز به مطرح شدن درکمیته اخلاق درپژوهش های علوم پزشکی دانشگاه را دارد؟

☑ بلی ( تاریخ جلسه: / / )

🗆 خیر

قسمت سوم-اطلاعات مربوط به روش اجرای طرح

**1-3 نوع مطالعه (*Type of Study*)**

🗆کیفی ☑کارآزمایی بالینی 🗆اپیدمیولوژیک تحلیلی(مورد شاهدی ،هم گروهی)

🗆تولیدی 🗆پایه (تجربی) 🗆اپیدمیولوژیک توصیفی 🗆مبتنی براطلاعات بیمارستانی

**2-3 روش اجرا وطراحی تحقیق  *Design*) *Research &* *Summary of Methodology*)**

این مطالعه كلینیكی بر روی كودكان 2 تا 12 ساله مراجعه كننده به درمانگاه اطفال بیمارستان كودكان قدس شهر قزوین انجام میشود. این كودكان به دلیل عفونت های تنفسی فوقانی و با علائم زیر مراجعه میكنند: وجود رینوره یا سرفه به مدت حداكثر 7 روز. سایر علائم میتوانند شامل موارد زیر باشند: احتقان بینی، تب كمتر از 39 درجه سانتی گراد، گلودرد، بی حالی و سردرد. بیمارانی كه علائم و نشانه های بیماری های مهم دستگاه تنفسی از جمله آسم، پنومونی، لارینژوتراكئوبرونشیت، سینوزیت و رینیت آلرژیك را دارا باشند از مطالعه حذف میشوند. همچنین، بیمارانی كه سابقه بیماری زمینه ای، بستری مكرر در بیمارستان یا مصرف دیفن‌هیدرامین در روزهای اخیر دارند، از مطالعه حذف میشوند. بیمارانی كه در طی دوره بیماری درمان آنالژزیك دریافت كرده باشند (نظیر استامینوفن یا ایبوبروفن) از مطالعه حذف نخواهند شد. در ابتدای مراجعه كودكان معاینه میشوند، سپس از تمام والدین خواسته میشود تا یك پرسش نامه 5 آیتمی را پر نمایند. در این پرسش‌نامه، والدین به طور سابژكتیو وضعیت سرفه كودك خود (به صورت دفعات سرفه، شدت سرفه و میزان آزاردهندگی سرفه) و كیفیت خواب كودك و نیز كیفیت خواب خودشان (از نظر میزان تاثیر سرفه شبانه كودك بر خواب وی و میزان كسر خواب والدین ناشی از سرفه كودك) در شبهای گذشته را ذكر و درجه بندی میكنند. این پرسش نامه از مطالعات انجام شده مشابه قبلی برداشته شده و مورد اعتبار است (6). در این پرسش نامه، برای هر آیتم بر اساس شدت، اعداد 0 تا 6 (به ترتیب از كم به زیاد) در نظر گرفته شده است. تنها كودكانی كه برای حداقل دو مورد از سه مورد زیر، نمره 3 به بالا را دریافت می كنند، در مطالعه باقی می مانند: دفعات سرفه های كودك، تاثیر آن بر خواب كودك و نیز تاثیر آن بر خواب والدین.

سپس كودكان به صورت تصادفی به 3 گروه تقسیم میشوند: گروه دریافت كننده عسل طبیعی منطقه اردبیل به همراه درمان محافظتی (گروه اول)، گروه دریافت كننده عسل طبیعی منطقه خراسان به همراه درمان محافظتی (گروه دوم) و گروه دریافت كننده دیفن هیدرامین به همراه درمان محافظتی (گروه سوم). گروه سوم گروه شاهد این مطالعه است. افراد حاضر در هر گروه نسبت به درمان خود كورسازی نمیشوند.

به افراد گروه اول یك ظرف پلاستیكی حاوی عسل طبیعی منطقه اردبیل داده میشود و والدین میبایست در شب اول و دوم پس از مراجعه، هر شب 2.5 میلی لیتر (براي كودكان 2 تا 6 سال) يا 5 ميلي ليتر (براي كودكان 6 تا 12 سال) از این عسل را 30 دقیقه قبل از خواب به كودك خود بدهند. به افراد گروه دوم یك ظرف پلاستیكی حاوی عسل طبیعی منطقه خراسان داده میشود و والدین میبایست در شب اول و دوم پس از مراجعه، هر شب 2.5 میلی لیتر (براي كودكان 2 تا 6 سال) يا 5 ميلي ليتر (براي كودكان 6 تا 12 سال) از این عسل را 30 دقیقه قبل از خواب به كودك خود بدهند. به افراد گروه سوم یك ظرف پلاستیكی حاوی شربت دیفن هیدرامین داده میشود و والدین میبایست در شب اول و دوم پس از مراجعه، هر شب 2.5 میلی لیتر (براي كودكان 2 تا 6 سال) يا 5 ميلي ليتر (براي كودكان 6 تا 12 سال) از شربت دیفن هیدرامین را 30 دقیقه قبل از خواب به كودك خود بدهند.

درمان محافظتی شامل قطره سدیم كلرید بینی، بخور آب گرم، شستشوی بینی، و استفاده از استامینوفن در صورت پایداری تب می باشد. به تمام والدین از نظر شرایط بیماری و نحوه شركت در مطالعه و پر كردن پرسشنامه و دادن داروی مورد نظر به كودكان و پایش وضعیت آنها توضیح داده میشود، سپس يك رضايتنامه كتبي جهت حضور در مطالعه توسط والدين پر مي شود و كاملا به آنها اطلاع رسانی میشود كه در صورت بروز علائم خطر نظیر بدتر شدن علائم و یا پیدایش علائم جدید، با مسئول مطالعه (مهدیه یوسف زنجانی) تماس بگیرند. در ابتدای روز سوم پس از مراجعه، یعنی پس از دادن دو دوز از داروی مورد نظر، با والدین تماس گرفته میشود و از آنها درخواست میشود تا همان سوالات پرسش‌نامه را مجددا پاسخ دهند. سپس دو پاسخ اولیه و ثانویه ای كه والدین به پرسش‌نامه داده اند با یكدیگر مقایسه میشود تا تاثیر این دو نوع عسل و دیفن هیدرامین بر سرفه شبانه كوكان و كیفیت خواب شبانه این كودكان و والدین آنها بررسی شود.

**3-3 جامعه مورد مطالعه و روش نمونه گیری(*Sampling Procedures*)**

كودكان 2 تا 12 ساله مراجعه كننده به درمانگاه اطفال بیمارستان قدس شهر قزوین با علائم زیر: وجود رینوره یا سرفه به مدت حداكثر 7 روز. بیمارانی كه علائم و نشانه های بیماری های مهم دستگاه تنفسی از جمله آسم، پنومونی، لارینژوتراكئوبرونشیت، سینوزیت و رینیت آلرژیك را دارا باشند از مطالعه حذف میشوند. همچنین، بیمارانی كه سابقه بیماری زمینه ای، بستری مكرر در بیمارستان یا مصرف دیفن‌هیدرامین در روزهای اخیر دارند، از مطالعه حذف میشوند. بیمارانی كه در طی دوره بیماری درمان آنالژزیك دریافت كرده باشند (نظیر استامینوفن یا ایبوبروفن) از مطالعه حذف نخواهند شد.

روش نمونه گیری: نمونه گیری از افراد در این طرح كه به صورت كارآزمایی بالینی انجام می شود به طور عمومی از مراجعین درمانگاه اطفال بیمارستان قدس كه واجد شرایط هستند صورت می گیرد و ورود افراد تحت پژوهش به گروه ها با استفاده از جدول اعداد تصادفی و به صورت رندوم انجام خواهد شد.

One Way ANOVA Power Analysis Std Dev Standard

Average Total of Means Deviation Effect

Power n k N Alpha Beta (Sm) (S) Size

0.82407 11.00 4 44 0.05000 0.17593 0.40 0.75 0.5378

References

Desu, M. M. and Raghavarao, D. 1990. Sample Size Methodology. Academic Press. New York.

Fleiss, Joseph L. 1986. The Design and Analysis of Clinical Experiments. John Wiley & Sons. New York.

Kirk, Roger E. 1982. Experimental Design: Procedures for the Behavioral Sciences. Brooks/Cole. Pacific Grove, California.

Report Definitions

Power is the probability of rejecting a false null hypothesis. It should be close to one.

n is the average group sample size.

k is the number of groups.

Total N is the total sample size of all groups combined.

Alpha is the probability of rejecting a true null hypothesis. It should be small.

Beta is the probability of accepting a false null hypothesis. It should be small.

Sm is the standard deviation of the group means under the alternative hypothesis.

Standard deviation is the within group standard deviation.

The Effect Size is the ratio of Sm to standard deviation.

Summary Statements

In a one-way ANOVA study, sample sizes of 11, 11, 11, and 11 are obtained from the 4 groups whose means are to be compared. The total sample of 44 subjects achieves 82% power to detect differences among the means versus the alternative of equal means using an F test with a 0.05000 significance level. The size of the variation in the means is represented by their standard deviation which is 0.40. The common standard deviation within a group is assumed to be 0.75.

Details when Alpha = 0.05000, Power = 0.82407, SM = 0.40, S = 0.75

Percent Deviation Ni

Ni of From Times

Group Ni Total Ni Mean Mean Deviation

1 11 25.00 1.89 0.37 4.04

2 11 25.00 1.89 0.37 4.04

3 11 25.00 1.39 0.13 1.46

4 11 25.00 0.92 0.60 6.63

ALL 44 100.00 1.52

One Way ANOVA Power Analysis

Multiple Comparisons Power Analysis

Numeric Results for Multiple Comparison Test: Dunnett (With Control)

Average Minimum Standard

Size Total Detectable Deviation

Power (n) k N Alpha Beta Difference (S) Diff / S

0.80016 29.00 4 116 0.05000 0.19984 1.00 0.75 1.3333

References

Hsu, Jason. 1996. Multiple Comparisons: Theory and Methods. Chapman & Hall. London.

Report Definitions

Power is the probability of rejecting a false null hypothesis. It should be close to one.

n is the average group sample size.

k is the number of groups.

Total N is the total sample size of all groups combined.

Alpha is the probability of rejecting a true null hypothesis. It should be small.

Beta is the probability of accepting a false null hypothesis. It should be small.

The Minimum Detectable Difference between any two group means.

S is the within group standard deviation.

Diff / D is the ratio of Min. Detect. Diff. to standard deviation.

Summary Statements

In a single factor ANOVA study, sample sizes of 29, 29, 29, and 29 are obtained from the 4 groups whose means are to be compared. The total sample of 116 subjects achieves 80% power to detect a difference of at least 1.00 using the Dunnett (With Control) multiple comparison test at a 0.05000 significance level. The common standard deviation within a group is assumed to be 0.75.

Dunnett Test Details

Percent Minimum

n of Detectable Standard

Group n Total N Alpha Power Difference Deviation

1 29 25.00 0.05000 0.80016 1.00 0.75

2 29 25.00

3 29 25.00

Control 29 25.00

Total 116 100.00

t tests - Means: Difference between two independent means (two groups)

Analysis: A priori: Compute required sample size

Input: Tail(s) = Two

Effect size d = 0.7872941

α err prob = 0.05

Power (1-β err prob) = 0.80

Allocation ratio N2/N1 = 1

Output: Noncentrality parameter δ = 2.8927032

Critical t = 2.0066468

Df = 52

Sample size group 1 = 27

Sample size group 2 = 27

Total sample size = 54

Actual power = 0.8102825

برای اثبات وجود اختلاف بین گروه ها در هر گروه تنها 11 نفر مورد نیاز است ولی برای این که بتوانیم در مقایسه گروه ها حداقل اختلاف 1 را نشان دهیم به 20-30 نفر در هر گروه نیاز خواهیم داشت.

**4-3 روش جمع آوری وتجزیه تحلیل داده ها (نام آزمون های آماری قید شود)**

داده ها با استفاده از نرم افزار آماری SPSS-19 مورد تجزیه و تحلیل قرار خواهد گرفت. برای توصیف داده ها از شاخص های مرکزی و پراکندگی شامل فراوانی، میانگین و انحراف معیار استفاده خواهد شد. برای مقایسه نتایج قبل و بعد از تجویز ماده موثره، از آزمون تی زوجی و برای مقایسه میزان تفاوت بین گروه ها از آزمون ANOVA، و برای مقایسه وجود ارتباط بین متغیرهای کیفی از آزمون کای دو استفاده خواهد شد. مقدار معنی دار Pکمتر از 05/0 در نظر گرفته خواهد شد.

**7-3 ملاحظات اخلاقی (*Ethical Review*)**

1- در همه گروه ها درمان محافظتی كه عموما در بیماری هاي ویرال درمان اصلی محسوب میشود ارائه میگردد و در گروه های مورد آزمایش، برای سرفه بیماران، عسل یا دیفن هیدرامین تجویز می شود.

2- درمان های معمول ضد سرفه با خطرات و عوارض جانبی همراه هستند و در سازمان های رسمی بهداشتی جهان از نظر تاثیر لازم و بیخطر بودن تایید نشده اند (6 و 9). دیفن‌هیدرامین یكی از این داروهاست كه اثرات موثر درمانی آن مورد بحث و جدل است (10 و 11). پس عدم تجویز آن در گروه هاي آزمايش از نظر سازمان های رسمی بهداشتی جهان مورد نكوهش قرار نخواهد گرفت.

3- افراد، با اطلاع كافی و توجیه كامل وارد این طرح میشوند و حضور آن ها در تمام مراحل انجام كار با رضایت كامل و کتبی آنان صورت خواهد گرفت و می توانند هر زمان كه بخواهند از مطالعه خارج شوند.

4- در تمام مراحل انجام طرح، بیماران، تحت كنترل و پایش مسئول پژوهش قرار دارند تا از بروز عارضه و خطر برای آنان جلوگیری شود.

**References**

6. Paul IM, Beiler J, McMonagle A, Shaffer ML, Duda L, Berlin CM Jr. Effect of honey dextromethorphan, and no treatment on nocturnal cough and sleep quality for coughing children and their parents. Arch Pediatr Adolesc Med. 2007;161(12):1140–1146.

9. Food and Drug Administration. FDA releases recommendations regarding use of over the-counter cough and cold products. January 17, 2008. Available at: [www.fda](http://www.fda/). gov/bbs/topics/NEWS/2008/NEW01778.html. Accessed May 12, 2011

10. Paul IM, Yoder KE, Crowell KR, et al. Effect of dextromethorphan, diphenhydramine, and placebo on nocturnal cough and sleep quality for coughing children and their parents. *Pediatrics*. 2004;114(1):e85-e90.

11. Bjornsdottir I, Einarson TR, Gudmundsson LS, Einarsdottir RA. Efficacy of diphenhydramine against cough in humans: A review. Pharm World Sci 2007;29:577–583.

**برگه اطلاع رسانی**

**عنوان پژوهش:**

**مقايسه اثر دو نوع عسل ايراني و ديفن هيدرامين بر سرفه شبانه كوكان و كيفيت خواب شبانه اين كودكان و والدين آنها**

عفونت هاي دستگاه تنفسي از بيماري هاي شايع در ميان كودكان است و هر ساله تعداد زيادي از آنها را راهي مطب پزشكان مينمايد. علائم شايع در عفونت هاي تنفسي كه اكثرا ويروسي هستند، شامل موارد زير است: آب ريزش از بيني، عطسه، ناخوشي، تب مختصر و سرفه. بيشتر علائم طي 3 روز اول شدت ميگيرند ولي خوشبختانه در مدت 1 هفته برطرف ميشوند، اما سرفه ممكن است تا مدت بيشتري باقي بماند. سرفه در كودكان ميتواند مشكلات زيادي براي آن ها و والدينشان ايجاد كند، به خصوص در شب، به دليل مشكلاتي كه در خواب كودك ايجاد ميكند، ميتواند به طور جدي آزاردهنده باشد و موجب كاهش ميزان خواب هم در كودكان مبتلا و هم در والدين آنها شود. در نتيجه، كودكان از امور درسي روزانه خود و والدين از فعاليت هاي روزانه خود به دليل خستگي بازمي مانند. در اين تحقيق ما بر آن شديم تا با بررسي روش هاي مناسب تر، گامي در جهت بهبود سرفه ناشي از عفونت هاي تنفسي در كودكان و متعاقبا بهبود كيفيت خواب آنها و والدينشان برداريم. در اين مطالعه از شما دعوت ميشود تا به طور داوطلبانه، تحت نظر پزشك فوق تخصص و نيز ساير كادر بهداشتي-درماني، وارد سير درماني مورد نظر شويد. در اين روند، به كودك شما به طور تصادفي يكي از انواع درمان (عسل یا شربت دیفن هیدرامین) ارائه ميشود. ممکن است هیچ هزینه ای جهت انجام این درمان ها از شما اخذ نخواهد شد. نتایج صرفا به صورت گروهی منتشر ميشود و اطلاعات شخصي شما محفوظ خواهد ماند. بدیهی است که شرکت شما در این پژوهش کاملا داوطلبانه بوده و درصورت عدم رضایت شما در هر مرحله ای که بخواهید می توانید انصراف خود را از ادامه همکاری اعلام نموده و پژوهش را ترک فرمایید. در صورت بروز هر مشكل و يا سوال و ابهامي ميتوانيد با مسئول پژوهش، خانم دكتر ‌**مهديه‌ يوسف ‌زنجاني** ، با شماره - تماس حاصل نماييد.

# **
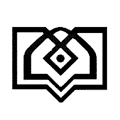
**

# **رضـايت نامـه**

**تمامي رضايت نامه ها بايد داراي امضاء و اثر انگشت مشخص بيمارمورد مطالعه باشد (در صورتي كه بيمار به سن قانوني نرسيده باشد و يا دچار اختلالات ذهني باشد رضايت نامه بايد توسط ولي يا سرپرست قانوني وي تكميل گردد).**

| پاسخ سوالات ذيل بايد توسط بيمار تكميل گردد | ***بلي*** | ***خير*** |
| --- | --- | --- |
| آيا برگه اطلاعاتي فرد مورد پژوهش را مطالعه و امضاء كرده ايد؟ |  |  |
| آيا فرصت پرسيدن سوال راجع به اين مطالعه و پژوهش يا بحث و تبادل نظر درباره آن را داشته ايد؟ |  |  |
| آيا براي تمامي سؤالات خود، جواب قانع كننده دريافت كرده ايد؟ |  |  |
| آيا درباره مطالعه، اطلاعات كافي به شما ارائه شده است؟ |  |  |
| مشاركت دراين پژوهش كاملا اختياري است و هرزمان كه بخواهيد بدون ارائه دليل مي توانيد كناره گيري نماييد. آيا از اين موضوع اطلاع داريد؟ |  |  |
| نام فردي كه در اين مورد با او صحبت كرده ايد را بنويسيد؟ | | |

بدينوسيله اينجانب ...................................... رضايت مي دهم كه به عنوان يك فرد مورد مطالعه در پژوهش " **مقايسه اثر دو نوع عسل ايراني و ديفن هيدرامين بر سرفه شبانه كوكان و كيفيت خواب شبانه اين كودكان و والدين آنها** " به سرپرستي خانم **مهديه يوسف زنجاني** شركت نمايم.

ممكن است اين تحقيق براي من فايده آني نداشته باشد ولي احتمالا براي ساير بيماران و رشد دانش پزشكي موثر خواهد بود.

كليه اطلاعاتي كه از من گرفته مي شود و نيز نام من محرمانه باقي خواهد ماند و نتايج تحقيقات به صورت كلي و در قالب اطلاعات گروه مورد مطالعه منتشر مي گردد و نتايج فردي در صورت نياز بدون ذكر نام و مشخصات فردي عرضه خواهد شد و همچنين برائت پزشك يا پزشكان اين طرح را از كليه اقدامات مذكور در برگه اطلاعاتي در صورت عدم تقصير در ارائه اقدامات اعلام مي دارم.

اين موافقت مانع از اقدامات قانوني اينجانب در مقابل دانشگاه، بيمارستان ، پژوهشگر و كارمندان در صورتي كه عملي خلاف و غير انساني انجام شود نخواهد بود.

نشاني وتلفني كه مي توان با بيمار تماس گرفت:

## **امضاء و اثر انگشت فرد مورد پژوهش: تاريخ:**

## **امضاء پژوهشگر:**

**برگ اطلاعات علمي پژوهشي**

سرپرست پژوهش:مهديه يوسف زنجاني، پرویز ایازی

موضوع پژوهش:

مقايسه اثر دو نوع عسل ايراني و ديفن هيدرامين بر سرفه شبانه كوكان و كيفيت خواب شبانه اين كودكان و والدين آنها

مدت پژوهش:2 سال

هدف پژوهش: معرفي ماده غذايي عسل به عنوان بهبود دهنده سرفه شبانه در كودكان

*(در صورتی که در هر مورد،رفرانس وجود دارد، آن را ذکر نمایید).*

اقداماتي كه براي انجام پژوهش بر روي فرد مورد مطالعه انجام مي شود:

تجويز عسل يا ديفن هيدرامين برای بيماران مبتلا به سرفه ناشي از عفونت ويروسي تنفسي

عوارض جانبي احتمالي:

عدم بهبود سرفه

فوائد احتمالي پژوهش:

بهبود سريع تر سرفه

فعاليت هايي كه در طول پژوهش بايد از آن اجتناب كرد:

مصرف داروهاي ديگر دور از نظر مسئول پژوهش و هر گونه ماده تحريك كننده مجاري تنفسي.

در صورت بروز هرگونه مشكل يا عارضه احتمالي مي توانيد با خانم مهديه يوسف زنجاني با شماره زیر تماس بگيريد:

امضاء و اثر انگشت بيمار : تاريخ:
