# Supplementary material for: Comparison of the Effect of Two Kinds of Iranian Honey and Diphenhydramine on Nocturnal Cough and the Sleep Quality in Coughing Children and Their Parents
Source: PLoS One. 2017 Jan 19;12(1):e0170277. doi: 10.1371/journal.pone.0170277 (PMC5245888; doi:10.1371/journal.pone.0170277)
Supplement: S3 File — (DOCX) [file pone.0170277.s003.docx]

**
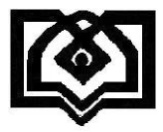
**

**In the name of God**

**Qazvin University of Medical Sciences**

**Deputy of Research and Technology**

**Proposal form**

**Title:**

Comparison of the Effect of Two Kinds of Iranian Honey and Diphenhydramine on Nocturnal Cough and Sleep Quality in Coughing Children and Their Parents

**Name and surname of project managers:**

Parviz Ayazi, Mahdieh Yousef-Zanjani

**Name of the school:**

School of Medicine

**Type of the study**:

Qualitative 🗆 Clinical trial ☑ Analytical epidemiologic (case-control, cohort) 🗆

Productive🗆 Basic (experimental) 🗆 Descriptive epidemiologic 🗆 Hospital-based 🗆

**Part Three- Information on Methods**

**3-1 Type of the study**

Qualitative 🗆 Clinical trial ☑ Analytical epidemiologic (case-control, cohort) 🗆

Productive🗆 Basic (experimental) 🗆 Descriptive epidemiologic 🗆 Hospital-based 🗆

**3-2 Methods**

This clinical trial will be performed on children aged 2-12 years presenting to the pediatric clinic of Qods Children’s Hospital in Qazvin, Iran. Those children suffer upper respiratory tract infection with the following symptoms: rhinorrhea or coughs up to 7 days. Other symptoms may be as follows: nasal congestion, fever lower than 39 °C, sore throat, malaise, and headache. Patients with signs and symptoms of important respiratory diseases, including asthma, pneumonia, laryngotracheobronchitis, sinusitis, and allergic rhinitis will be excluded from the study. Furthermore, patients with a history of underlying diseases, frequent hospitalizations, or use of diphenhydramine in recent days will be excluded from the study. Patients who have received analgesics during the course of a disease (e.g., acetaminophen or ibuprofen) will not be excluded from the study. The children will be examined upon admission, and then, all parents will be requested to complete a 5-item inventory. Parents subjectively mention and rate their child’s coughing (frequency, severity, and irritability of coughs), child’s quality of sleeping, and quality of parents’ sleeping (to what degree the child’s overnight coughing affects his own sleeping and reduces his parents’ sleeping) over previous nights in the inventory. The inventory has been adopted from previous studies and is valid (6). Each item of the inventory is assigned a point from 0 to 6 (respectively from low to high) based on its intensity. Only children gaining scores ≥ 3 for at least two items of the following three items will remain in the study: frequency of coughs, impact of coughs on child's sleeping, and impact of child’s coughs on parents’ sleeping.

The children will randomly be divided into 3 groups: a group receiving natural honey produced in Ardebil, Iran along with conservative treatment (the first group), a group receiving natural honey produced in Khorasan, Iran along with conservative treatment(the second group), and a group receiving diphenhydramine along with conservative treatment(the third group). The third group is the control group of this study. Patients in each group will not be blinded for their treatment.

Patients in the first group will receive a plastic container containing natural honey produced in Ardebil, and their parents should give 2.5 ml (for children aged 2-6 years) or 5 ml (for children aged 6-12 years) of honey to their child 30 minutes before child’s sleeping on the first and second nights after going to the clinic. Patients in the second group will receive a plastic container containing natural honey produced in Khorasan, and their parents should give 2.5 ml (for children aged 2-6 years) or 5 ml (for children aged 6-12 years) of honey to their child 30 minutes before child’s sleeping on the first and second nights after going to the clinic. Patients in the third group will receive a plastic container containing diphenhydramine syrup, and their parents should give 2.5 ml (for children aged 2-6 years) or 5 ml (for children aged 6-12 years) of the syrup to their child 30 minutes before child’s sleeping on the first and second nights after going to the clinic.

The conservative treatment includes nasal sodium chloride drops, steam inhalation, nasal wash, and use of acetaminophen if fever persists. Parents will be briefed on the disease conditions, how to participate in the study,how to complete the inventory, how to administer the drug to children, and how to monitor them. Parents will then complete a written consent for their participation in the study and will be asked to contact the corresponding author (Mahdieh Yousef-Zanjani) in case of warning symptoms, such as exacerbation of symptoms or appearance of new symptoms. The researcher will call the parents at the beginning of the third day, after giving two doses of the relevant drug to children, and request them to complete the same inventory again. Then, the first and the second answers given by the parents will be compared in order to examine the effect of the two types of honey and diphenhydramine on children's overnight coughs and quality of night sleeping in children and their parents.

**3-3 Study population and sampling method**

The study population includes children aged 2-12 yearspresenting to the pediatric clinic of Qods Children’s Hospital in Qazvin with rhinorrhea or coughs lasting up to 7 days. Patients with symptoms of important respiratory tract diseases, including asthma, pneumonia, laryngotracheobronchitis, sinusitis, and allergic rhinitis will be excluded from the study. Moreover, patients with a history of underlying diseases, frequent hospitalizations, or use of diphenhydramine in recent days will be excluded from the study. Patients who have received analgesic therapy during the course of a disease (e.g., acetaminophen or ibuprofen) will not be excluded from the study.

Sampling method: Patients in this clinical trial will be selected generally from patients going to the pediatric clinic of Qods Children’s Hospital with inclusion criteria. The patients will randomly be assigned to the groups using the table of random numbers.

One Way ANOVA Power Analysis Std Dev Standard

Average Total of Means Deviation Effect

Power n k N Alpha Beta (Sm) (S) Size

0.82407 11.00 4 44 0.05000 0.17593 0.40 0.75 0.5378

References

Desu, M. M. and Raghavarao, D. 1990. Sample Size Methodology. Academic Press. New York.

Fleiss, Joseph L. 1986. The Design and Analysis of Clinical Experiments. John Wiley & Sons. New York.

Kirk, Roger E. 1982. Experimental Design: Procedures for the Behavioral Sciences. Brooks/Cole. Pacific Grove, California.

Report Definitions

Power is the probability of rejecting a false null hypothesis. It should be close to one.

n is the average group sample size.

k is the number of groups.

Total N is the total sample size of all groups combined.

Alpha is the probability of rejecting a true null hypothesis. It should be small.

Beta is the probability of accepting a false null hypothesis. It should be small.

Sm is the standard deviation of the group means under the alternative hypothesis.

Standard deviation is the within group standard deviation.

The Effect Size is the ratio of Sm to standard deviation.

Summary Statements

In a one-way ANOVA study, sample sizes of 11, 11, 11, and 11 are obtained from the 4 groups whose means are to be compared. The total sample of 44 subjects achieves 82% power to detect differences among the means versus the alternative of equal means using an F test with a 0.05000 significance level. The size of the variation in the means is represented by their standard deviation which is 0.40. The common standard deviation within a group is assumed to be 0.75.

Details when Alpha = 0.05000, Power = 0.82407, SM = 0.40, S = 0.75

Percent Deviation Ni

Ni of From Times

Group Ni Total Ni Mean Mean Deviation

1 11 25.00 1.89 0.37 4.04

2 11 25.00 1.89 0.37 4.04

3 11 25.00 1.39 0.13 1.46

4 11 25.00 0.92 0.60 6.63

ALL 44 100.00 1.52

One Way ANOVA Power Analysis

Multiple Comparisons Power Analysis

Numeric Results for Multiple Comparison Test: Dunnett (With Control)

Average Minimum Standard

Size Total Detectable Deviation

Power (n) k N Alpha Beta Difference (S) Diff / S

0.80016 29.00 4 116 0.05000 0.19984 1.00 0.75 1.3333

References

Hsu, Jason. 1996. Multiple Comparisons: Theory and Methods. Chapman & Hall. London.

Report Definitions

Power is the probability of rejecting a false null hypothesis. It should be close to one.

n is the average group sample size.

k is the number of groups.

Total N is the total sample size of all groups combined.

Alpha is the probability of rejecting a true null hypothesis. It should be small.

Beta is the probability of accepting a false null hypothesis. It should be small.

The Minimum Detectable Difference between any two group means.

S is the within group standard deviation.

Diff / D is the ratio of Min. Detect. Diff. to standard deviation.

Summary Statements

In a single factor ANOVA study, sample sizes of 29, 29, 29, and 29 are obtained from the 4 groups whose means are to be compared. The total sample of 116 subjects achieves 80% power to detect a difference of at least 1.00 using the Dunnett (With Control) multiple comparison test at a 0.05000 significance level. The common standard deviation within a group is assumed to be 0.75.

Dunnett Test Details

Percent Minimum

n of Detectable Standard

Group n Total N Alpha Power Difference Deviation

1 29 25.00 0.05000 0.80016 1.00 0.75

2 29 25.00

3 29 25.00

Control 29 25.00

Total 116 100.00

t tests - Means: Difference between two independent means (two groups)

Analysis: A priori: Compute required sample size

Input: Tail(s) = Two

Effect size d = 0.7872941

α err prob = 0.05

Power (1-β err prob) = 0.80

Allocation ratio N2/N1 = 1

Output: Noncentrality parameter δ = 2.8927032

Critical t = 2.0066468

Df = 52

Sample size group 1 = 27

Sample size group 2 = 27

Total sample size = 54

Actual power = 0.8102825

Only 11 patients are needed for each group in order to prove the difference among groups, but 20-30 patients are needed in each group to be able to show minimum difference of 1 between every two groups.

**3-4 Data collection and analysis procedures (Name the statistical tests)**

The data will be analyzed using SPSS-19 software. Measures of central tendency and distribution, including frequency, mean, and standard deviation will be used to describe results. Results before and after administration of the relevant substance will be compared using paired *t* test, and the level of difference between groups will be examined using ANOVA. Chi-square test will be used to compare correlations between qualitative variables. P values less than 0.05 will be significant.

**3-7 Ethical considerations**

1-All groups will receive conservative treatment that is generally considered the main treatment of viral diseases, and honey or diphenhydramine is administered to the study groups to treat their coughing.

2- Usual cough-suppressing treatments are accompanied with side effects and risks and have not been confirmed by global official health organizations in terms of effectiveness and safety (6 & 9). Diphenhydramine is among drugs whose positive therapeutic effects are controversial (10 & 11). Therefore, global official health organizations will not criticize non-administration of diphenhydramine in the experimental groups.

3-Participants will participate in this study upon being completely informed and will be present in each stage of the study with their thorough and written consent. They can withdraw from the study whenever they want.

4- The corresponding author will control and monitor the patients in all procedures of the study in order to prevent any complications or risks.

**References**

6. Paul IM, Beiler J, McMonagle A, Shaffer ML, Duda L, Berlin CM Jr. Effect of honey dextromethorphan, and no treatment on nocturnal cough and sleep quality for coughing children and their parents. Arch Pediatr Adolesc Med. 2007;161(12):1140–1146.

9. Food and Drug Administration. FDA releases recommendations regarding use of over the-counter cough and cold products. January 17, 2008. Available at: [www.fda](http://www.fda). gov/bbs/topics/NEWS/2008/NEW01778.html. Accessed May 12, 2011

10. Paul IM, Yoder KE, Crowell KR, et al. Effect of dextromethorphan, diphenhydramine, and placebo on nocturnal cough and sleep quality for coughing children and their parents. *Pediatrics*. 2004;114(1):e85-e90.

11. Bjornsdottir I, Einarson TR, Gudmundsson LS, Einarsdottir RA. Efficacy of diphenhydramine against cough in humans: A review. Pharm World Sci 2007;29:577–583.

**Information sheet**

**Title:** Comparison of the Effect of Two Kinds of Iranian Honey and Diphenhydramine on Nocturnal Cough and Sleep Quality in Coughing Children and Their Parents

Respiratory tract infections are common in children, many of whom visit doctors for these infections every year. Common symptoms of respiratory infections that are mostly viral are as follows: rhinorrhea, sneezing, malaise, mild fever, and coughing. Most symptoms exacerbate during the first 3 days, but fortunately disappear within 1 week. However, coughs may remain longer. Coughing may cause many problems for children and their parents, especially at nights. Sleeping problems caused by coughing may be seriously annoying and reduce sleeping both in children themselves and their parents. In this respect, children’s daily assignments and parents’ daily activities are disturbed due to their fatigue. In this study, the researcher tries to use more efficient methods to suppress coughs caused by respiratory infections in children and consequently improve the quality of sleeping in children and their parents. You are kindly invited to enter the course of treatment in this study voluntarily under the supervision of a subspecialist and also the healthcare personnel. In this study, your child will randomly receive one of the treatments (honey or diphenhydramine syrup). These treatments will be delivered free of charge. Results will be published collectively, and your personal information will be confidential. Certainly, your participation in this study is on voluntary basis and you are able to withdraw from the study at any stage upon declaring your unwillingness to continue. You can contact the corresponding author, Dr Mahdieh Yousef-Zanjani, at ………… if you have any problems or questions.

**Consent form**

| **All consents should have the patient’s signature and clear fingerprint (if the patient has not reached the legal age or suffers mental disorders, the consent should be completed by his parent or legal guardian).** |
| --- |

| **The following items should be completed by the patient[/their parent or guardian]** | **Yes** | **No** |
| --- | --- | --- |
| Have you read and signed the participant’s information form? |  |  |
| Have you had any chance to ask about this study or discuss it with others? |  |  |
| Have you received convincing answers for all your questions? |  |  |
| Have you received adequate information about the study? |  |  |
| Participation in this study is quite voluntarily and you can withdraw from the study whenever you want without mentioning any reasons. Do you know this? |  |  |
| Mention the name of the person to whom you have talked about this study. | | |

I, ………………………., hereby declare my consent for participating in the study “**Comparison of the Effect of Two Kinds of Iranian Honey and Diphenhydramine on Nocturnal Cough and Sleep Quality in Coughing Children and Their Parents**” conducted by **Mahdieh Yousef-Zanjani**.

This study may not have an immediate advantage for me, but it will be likely to be effective for other patients and development of medicine.

All my information, including my name, will remain confidential, results of the study will be published generally and collectively, and any individual results will be presented without mentioning the name and personal information. Moreover, I declare physician(s) of this study are not liable for any actions mentioned in the information form if they do not occur due to their negligence.

This consent will not bar me from taking legal actions against the university, hospital, researcher, and personnel in case of any illegal and inhumane acts.

Patient’s phone number and address:

Participant’s signature and fingerprint: Date:

Researcher’s signature:

**Scientific - research information form**

| Corresponding author: Mahdieh Yousef-Zanjani, Parviz Ayazi  Title of the study:  Comparison of the Effect of Two Kinds of Iranian Honey and Diphenhydramine on Nocturnal Cough and Sleep Quality in Coughing Children and Their Parents  Duration of the study: 2 years  Objective of the study: Introduction of honey as a suppressor of children's overnight coughs |
| --- |

*(Mention the reference for each item if there is any reference.)*

**Actions that will be performed on participants are as follows:**

Administration of honey or diphenhydramine to patients coughing because of respiratory viral infections

**Possible side effects:**

Non-improvement of coughs

**Possible advantages of the study:**

Faster improvement of coughs

**Activities that should be avoided during the study are as follows:**

Administration of other drugs and any respiratory tract irritating substance without informing the corresponding author.

You can contact Mrs. Mahdieh Yousef Zanjani at ………… if any problems or likely complications appear.

Patient’s signature and fingerprint: Date:
