# Supplementary material for: Comparison of the Effect of Two Kinds of Iranian Honey and Diphenhydramine on Nocturnal Cough and the Sleep Quality in Coughing Children and Their Parents
Source: PLoS One. 2017 Jan 19;12(1):e0170277. doi: 10.1371/journal.pone.0170277 (PMC5245888; doi:10.1371/journal.pone.0170277)
Supplement: S5 File — (PDF) [file pone.0170277.s005.pdf]

```
GET
  FILE='C:\Users\Admin_pc\Desktop\Honey\Honey SPSS\Honey Data.sav'.

Warning # 67.  Command name: GET FILE
The document is already in use by another user or process.  If you make
changes to the document they may overwrite changes made by others or your
changes may be overwritten by others.
File opened C:\Users\Admin_pc\Desktop\Honey\Honey SPSS\Honey Data.sav
DATASET NAME DataSet1 WINDOW=FRONT.
DATASET ACTIVATE DataSet1.
DISPLAY DICTIONARY.
```

File Information

```
[DataSet1] C:\Users\Admin_pc\Desktop\Honey\Honey SPSS\Honey Data.sav
```

Variable Information

Variable	Position	Label	Measurement Level	Role	Column Width	Alignment	Print Format	Write Format
group	1	group	Scale	Input	8	Right	F8	F8
sex	2	sex	Scale	Input	8	Right	F8	F8
age	3	age	Scale	Input	8	Right	F8.1	F8.1
weight	4	weight	Scale	Input	8	Right	F8.1	F8.1
time	5	duration of the URTIs before enrollment	Scale	Input	8	Right	F8	F8
before_1	6	1. How frequent was your child’s coughing last night?	Scale	Input	8	Right	F8	F8

before_2		7	2. How severe was your child’s cough last night?	Scale	Input	8	Right	F8	F8
before_3		8	3. How bothersome was last night’s cough to your child?	Scale	Input	8	Right	F8	F8
before_4		9	4. How much did last night’s cough affect your child’s ability to sleep?	Scale	Input	8	Right	F8	F8
before_5		10	5. How much did last night’s cough affect your (parent’s) ability to sleep?	Scale	Input	8	Right	F8	F8
after_1		11	1. How frequent was your child’s coughing last night?	Scale	Input	8	Right	F8	F8
after_2		12	2. How severe was your child’s cough last night?	Scale	Input	8	Right	F8	F8

after_3	13	3. How bothersome was last night's cough to your child?	Scale	Input	8	Right	F8	F8
after_4	14	4. How much did last night's cough affect your child's ability to sleep?	Scale	Input	8	Right	F8	F8
after_5	15	5. How much did last night's cough affect your (parent's) ability to sleep?	Scale	Input	8	Right	F8	F8
before_total	16	before_total	Scale	Input	10	Right	F8.2	F8.2
after_total	17	after_total	Scale	Input	10	Right	F8.2	F8.2
Difference_1	18	Difference_1	Scale	Input	10	Right	F8.5	F8.5
Difference_2	19	Difference_2	Scale	Input	10	Right	F8.5	F8.5
Difference_3	20	Difference_3	Scale	Input	10	Right	F8.5	F8.5
Difference_4	21	Difference_4	Scale	Input	10	Right	F8.5	F8.5
Difference_5	22	Difference_5	Scale	Input	10	Right	F8.5	F8.5
Difference_total	23	Difference_total	Scale	Input	10	Right	F8.5	F8.5

Variables in the working file

Variable Values

Value		Label
group	1	honey-Kimia
	2	Diphenhydramine
	3	honey_Shahd-eGolha
sex	1	boy
	2	girl
