# Supplementary material for: Comparison of the Effect of Two Kinds of Iranian Honey and Diphenhydramine on Nocturnal Cough and the Sleep Quality in Coughing Children and Their Parents
Source: PLoS One. 2017 Jan 19;12(1):e0170277. doi: 10.1371/journal.pone.0170277 (PMC5245888; doi:10.1371/journal.pone.0170277)
Supplement: S6 File — (PDF) [file pone.0170277.s006.pdf]

```
GET
  FILE='F:\DrE,Qods\Dr Ayazi\Honey\Submit\PlosOne\95.5\Resubmit\Honey Data.sav'.
DATASET NAME DataSet0 WINDOW=FRONT.
DESCRIPTIVES VARIABLES=age

  /STATISTICS=MEAN STDDEV MIN MAX.
```

Descriptives

Notes

Output Created		10-Sep-2016 13:40:49
Comments		
Input	Data	F:\DrE,Qods\Dr Ayazi\Honey\Submit\PlosOne\95.5\Resubmit\Honey Data.sav
	Active Dataset	DataSet1
	Filter	<none>
	Weight	<none>
	Split File	<none>
	N of Rows in Working Data File	87
Missing Value Handling	Definition of Missing	User defined missing values are treated as missing.
	Cases Used	All non-missing data are used.
Syntax		DESCRIPTIVES VARIABLES=age /STATISTICS=MEAN STDDEV MIN MAX.
Resources	Processor Time	00:00:00.000
	Elapsed Time	00:00:00.000

```
[DataSet1] F:\DrE,Qods\Dr Ayazi\Honey\Submit\PlosOne\95.5\Resubmit\Honey Data.sav
```

Descriptive Statistics

	N	Minimum	Maximum	Mean	Std. Deviation
age	87	1.00	8.00	3.5287	1.58737
Valid N (listwise)	87				

```
ONEWAY age BY group

  /MISSING ANALYSIS.
```

Oneway

Notes

Output Created		10-Sep-2016 13:41:11
Comments		
Input	Data	F:\DrE,Qods\Dr Ayazi\Honey\Submit\PlosOne\95.5\Resubmit\Honey Data.sav
	Active Dataset	DataSet1
	Filter	<none>
	Weight	<none>
	Split File	<none>
	N of Rows in Working Data File	87
Missing Value Handling	Definition of Missing	User-defined missing values are treated as missing.
	Cases Used	Statistics for each analysis are based on cases with no missing data for any variable in the analysis.
Syntax		ONEWAY age BY group /MISSING ANALYSIS.
Resources	Processor Time	00:00:00.000
	Elapsed Time	00:00:00.000

```
[DataSet1] F:\DrE,Qods\Dr Ayazi\Honey\Submit\PlosOne\95.5\Resubmit\Honey Data.sav
```

ANOVA

age

	Sum of Squares	df	Mean Square	F	Sig.
Between Groups	10.488	2	5.244	2.136	.124
Within Groups	206.210	84	2.455		
Total	216.698	86			

```
FREQUENCIES VARIABLES=sex
  /ORDER=ANALYSIS.
```

Frequencies

Notes

Output Created		10-Sep-2016 13:41:37
Comments		
Input	Data	F:\DrE,Qods\Dr Ayazi\Honey\Submit\PlosOne\95.5\Resubmit\Honey Data.sav
	Active Dataset	DataSet1
	Filter	<none>
	Weight	<none>
	Split File	<none>
	N of Rows in Working Data File	87
Missing Value Handling	Definition of Missing	User-defined missing values are treated as missing.
	Cases Used	Statistics are based on all cases with valid data.
Syntax		FREQUENCIES VARIABLES=sex /ORDER=ANALYSIS.
Resources	Processor Time	00:00:00.000
	Elapsed Time	00:00:00.000

```
[DataSet1] F:\DrE,Qods\Dr Ayazi\Honey\Submit\PlosOne\95.5\Resubmit\Honey Data.sav
```

Statistics

sex

N	Valid	87
	Missing	0

sex

		Frequency	Percent	Valid Percent	Cumulative Percent
Valid	male	41	47.1	47.1	47.1
	female	46	52.9	52.9	100.0
	Total	87	100.0	100.0	

```
DESCRIPTIVES VARIABLES=time
  /STATISTICS=MEAN STDDEV MIN MAX.
```

Descriptives

Notes

Output Created		10-Sep-2016 13:42:04
Comments		
Input	Data	F:\DrE,Qods\Dr Ayazi\Honey\Submit\PlosOne\95.5\Resubmit\Honey Data.sav
	Active Dataset	DataSet1
	Filter	<none>
	Weight	<none>
	Split File	<none>
	N of Rows in Working Data File	87
Missing Value Handling	Definition of Missing	User defined missing values are treated as missing.
	Cases Used	All non-missing data are used.
Syntax		DESCRIPTIVES VARIABLES=time /STATISTICS=MEAN STDDEV MIN MAX.
Resources	Processor Time	00:00:00.000
	Elapsed Time	00:00:00.002

```
[DataSet1] F:\DrE,Qods\Dr Ayazi\Honey\Submit\PlosOne\95.5\Resubmit\Honey Data.sav
```

Descriptive Statistics

	N	Minimum	Maximum	Mean	Std. Deviation
duration of the URTIs before enrollment	87	1	7	3.49	2.134
Valid N (listwise)	87				

```
ONEWAY time BY group
  /MISSING ANALYSIS.
```

Oneway

Notes

Output Created		10-Sep-2016 13:42:53
Comments		
Input	Data	F:\DrE,Qods\Dr Ayazi\Honey\Submit\PlosOne\95.5\Resubmit\Honey Data.sav
	Active Dataset	DataSet1
	Filter	<none>
	Weight	<none>
	Split File	<none>
	N of Rows in Working Data File	87
Missing Value Handling	Definition of Missing	User-defined missing values are treated as missing.
	Cases Used	Statistics for each analysis are based on cases with no missing data for any variable in the analysis.
Syntax		ONEWAY time BY group /MISSING ANALYSIS.
Resources	Processor Time	00:00:00.000
	Elapsed Time	00:00:00.000

```
[DataSet1] F:\DrE,Qods\Dr Ayazi\Honey\Submit\PlosOne\95.5\Resubmit\Honey Data.sav
```

ANOVA

duration of the URTIs before enrollment

	Sum of Squares	df	Mean Square	F	Sig.
Between Groups	9.482	2	4.741	1.042	.357
Within Groups	382.265	84	4.551		
Total	391.747	86			

```
CROSSTABS
  /TABLES=group BY sex
  /FORMAT=AVALUE TABLES
  /STATISTICS=CHISQ
  /CELLS=COUNT ROW

  /COUNT ROUND CELL.
```

Crosstabs

Notes

Output Created		10-Sep-2016 13:43:39
Comments		
Input	Data	F:\DrE,Qods\Dr Ayazi\Honey\Submit\PlosOne\95.5\Resubmit\Honey Data.sav
	Active Dataset	DataSet1
	Filter	<none>
	Weight	<none>
	Split File	<none>
	N of Rows in Working Data File	87
Missing Value Handling	Definition of Missing	User-defined missing values are treated as missing.
	Cases Used	Statistics for each table are based on all the cases with valid data in the specified range(s) for all variables in each table.
Syntax		CROSSTABS<br>/TABLES=group BY sex<br>/FORMAT=AVALUE TABLES<br>/STATISTICS=CHISQ<br>/CELLS=COUNT ROW<br>/COUNT ROUND CELL.
Resources	Processor Time	00:00:00.000
	Elapsed Time	00:00:00.000
	Dimensions Requested	2
	Cells Available	174762

```
[DataSet1] F:\DrE,Qods\Dr Ayazi\Honey\Submit\PlosOne\95.5\Resubmit\Honey Data.sav
```

Case Processing Summary

	Cases					
	Valid		Missing		Total	
	N	Percent	N	Percent	N	Percent
group * sex	87	100.0%	0	.0%	87	100.0%

group * sex Crosstabulation

			sex		Total
			male	female	
group	honey-Kimia	Count	20	22	42
		% within group	47.6%	52.4%	100.0%
	Diphenhydramine	Count	7	13	20
		% within group	35.0%	65.0%	100.0%
	honey_Shahd-eGolha	Count	14	11	25
		% within group	56.0%	44.0%	100.0%
Total		Count	41	46	87
		% within group	47.1%	52.9%	100.0%

Chi-Square Tests

	Value	df	Asymp. Sig. (2-sided)
Pearson Chi-Square	1.974[a]	2	.373
Likelihood Ratio	1.997	2	.368
Linear-by-Linear Association	.252	1	.616
N of Valid Cases	87		

a. 0 cells (.0%) have expected count less than 5. The minimum expected count is 9.43.

```
GLM before_1 before_2 before_3 before_4 before_5 before_total BY group WITH age
  /METHOD=SSTYPE(3)
  /INTERCEPT=INCLUDE
  /EMMEANS=TABLES(group) WITH(age=MEAN) COMPARE ADJ(LSD)
  /PRINT=DESCRIPTIVE
  /CRITERIA=ALPHA(.05)

  /DESIGN=age group.
```

General Linear Model

Notes

Output Created		10-Sep-2016 13:44:44
Comments		
Input	Data	F:\DrE,Qods\Dr Ayazi\Honey\Submit\PlosOne\95.5\Resubmit\Honey Data.sav

	Active Dataset	DataSet1
	Filter	<none>
	Weight	<none>
	Split File	<none>
	N of Rows in Working Data File	87
Missing Value Handling	Definition of Missing	User-defined missing values are treated as missing.
	Cases Used	Statistics are based on all cases with valid data for all variables in the model.
Syntax		GLM before_1 before_2 before_3 before_4 before_5 before_total BY group WITH age<br>/METHOD=SSTYPE(3)<br>/INTERCEPT=INCLUDE<br>/EMMEANS=TABLES(group) WITH(age=MEAN) COMPARE ADJ(LSD)<br>/PRINT=DESCRIPTIVE<br>/CRITERIA=ALPHA(.05)<br>/DESIGN=age group.
Resources	Processor Time	00:00:00.016
	Elapsed Time	00:00:00.031

[DataSet1] F:\DrE,Qods\Dr Ayazi\Honey\Submit\PlosOne\95.5\Resubmit\Honey Data.sav

Between-Subjects Factors

		Value Label	N
group	1	honey-Kimia	42
	2	Diphenhydramine	20
	3	honey_Shahd-e Golha	25

Descriptive Statistics

	group	Mean	Std. Deviation	N
1. How frequent was your child's coughing last night?	honey-Kimia	3.38	.936	42
	Diphenhydramine	3.45	1.191	20
	honey_Shahd-eGolha	3.72	.792	25

	Total	3.49	.963	87
2. How severe was your child's cough last night?	honey-Kimia	3.74	.828	42
	Diphenhydramine	3.65	.933	20
	honey_Shahd-eGolha	4.20	.707	25
	Total	3.85	.843	87
3. How bothersome was last night's cough to your child?	honey-Kimia	3.60	.885	42
	Diphenhydramine	2.85	.875	20
	honey_Shahd-eGolha	3.56	.821	25
	Total	3.41	.909	87
4. How much did last night's cough affect your child's ability to sleep?	honey-Kimia	3.14	1.026	42
	Diphenhydramine	2.45	1.099	20
	honey_Shahd-eGolha	3.40	1.291	25
	Total	3.06	1.165	87
5. How much did last night's cough affect your (parent's) ability to sleep?	honey-Kimia	3.67	.816	42
	Diphenhydramine	2.65	.933	20
	honey_Shahd-eGolha	4.20	.707	25
	Total	3.59	.983	87
before_total	honey-Kimia	17.5238	3.14090	42
	Diphenhydramine	15.0500	4.43046	20
	honey_Shahd-eGolha	19.0800	3.91493	25
	Total	17.4023	3.92801	87

Multivariate Tests[c]

Effect		Value	F	Hypothesis df	Error df	Sig.
Intercept	Pillai's Trace	.791	59.831[a]	5.000	79.000	.000
	Wilks' Lambda	.209	59.831[a]	5.000	79.000	.000
	Hotelling's Trace	3.787	59.831[a]	5.000	79.000	.000
	Roy's Largest Root	3.787	59.831[a]	5.000	79.000	.000
age	Pillai's Trace	.101	1.773[a]	5.000	79.000	.128
	Wilks' Lambda	.899	1.773[a]	5.000	79.000	.128
	Hotelling's Trace	.112	1.773[a]	5.000	79.000	.128
	Roy's Largest Root	.112	1.773[a]	5.000	79.000	.128
group	Pillai's Trace	.556	6.155	10.000	160.000	.000
	Wilks' Lambda	.510	6.331[a]	10.000	158.000	.000

	Hotelling's Trace	.834	6.504	10.000	156.000	.000
	Roy's Largest Root	.631	10.089[b]	5.000	80.000	.000

a. Exact statistic

b. The statistic is an upper bound on F that yields a lower bound on the significance level.

c. Design: Intercept + age + group

Tests of Between-Subjects Effects

Source	Dependent Variable	Type III Sum of Squares	df	Mean Square	F	Sig.
Corrected Model	1. How frequent was your child's coughing last night?	2.378[a]	3	.793	.850	.470
	2. How severe was your child's cough last night?	7.500[b]	3	2.500	3.874	.012
	3. How bothersome was last night's cough to your child?	11.444[c]	3	3.815	5.307	.002
	4. How much did last night's cough affect your child's ability to sleep?	12.616[d]	3	4.205	3.353	.023
	5. How much did last night's cough affect your (parent's) ability to sleep?	30.446[e]	3	10.149	15.997	.000
	before_total	237.576[f]	3	79.192	6.034	.001
Intercept	1. How frequent was your child's coughing last night?	160.806	1	160.806	172.508	.000
	2. How severe was your child's cough last night?	170.353	1	170.353	264.000	.000
	3. How bothersome was last night's cough to your child?	121.800	1	121.800	169.451	.000
	4. How much did last night's cough affect your child's ability to sleep?	101.785	1	101.785	81.157	.000
	5. How much did last night's cough affect your (parent's) ability to sleep?	136.151	1	136.151	214.607	.000
	before_total	3425.341	1	3425.341	260.986	.000
age	1. How frequent was your child's coughing last night?	.525	1	.525	.563	.455

	2. How severe was your child's cough last night?	3.111	1	3.111	4.821	.031
	3. How bothersome was last night's cough to your child?	3.170	1	3.170	4.410	.039
	4. How much did last night's cough affect your child's ability to sleep?	1.997	1	1.997	1.592	.211
	5. How much did last night's cough affect your (parent's) ability to sleep?	3.226	1	3.226	5.085	.027
	before_total	55.922	1	55.922	4.261	.042
group	1. How frequent was your child's coughing last night?	2.147	2	1.074	1.152	.321
	2. How severe was your child's cough last night?	5.271	2	2.636	4.085	.020
	3. How bothersome was last night's cough to your child?	7.076	2	3.538	4.922	.010
	4. How much did last night's cough affect your child's ability to sleep?	10.248	2	5.124	4.085	.020
	5. How much did last night's cough affect your (parent's) ability to sleep?	26.845	2	13.423	21.157	.000
	before_total	181.336	2	90.668	6.908	.002
Error	1. How frequent was your child's coughing last night?	77.370	83	.932		
	2. How severe was your child's cough last night?	53.558	83	.645		
	3. How bothersome was last night's cough to your child?	59.659	83	.719		
	4. How much did last night's cough affect your child's ability to sleep?	104.096	83	1.254		
	5. How much did last night's cough affect your (parent's) ability to sleep?	52.657	83	.634		
	before_total	1089.344	83	13.125		
Total	1. How frequent was your child's coughing last night?	1142.000	87			

	2. How severe was your child's cough last night?	1351.000	87			
	3. How bothersome was last night's cough to your child?	1085.000	87			
	4. How much did last night's cough affect your child's ability to sleep?	930.000	87			
	5. How much did last night's cough affect your (parent's) ability to sleep?	1202.000	87			
	before_total	27674.000	87			
Corrected Total	1. How frequent was your child's coughing last night?	79.747	86			
	2. How severe was your child's cough last night?	61.057	86			
	3. How bothersome was last night's cough to your child?	71.103	86			
	4. How much did last night's cough affect your child's ability to sleep?	116.713	86			
	5. How much did last night's cough affect your (parent's) ability to sleep?	83.103	86			
	before_total	1326.920	86			

a. R Squared = .030 (Adjusted R Squared = -.005)

b. R Squared = .123 (Adjusted R Squared = .091)

c. R Squared = .161 (Adjusted R Squared = .131)

d. R Squared = .108 (Adjusted R Squared = .076)

e. R Squared = .366 (Adjusted R Squared = .343)

f. R Squared = .179 (Adjusted R Squared = .149)

Estimated Marginal Means

group

Estimates

				95% Confidence Interval	
Dependent Variable	group	Mean	Std. Error	Lower Bound	Upper Bound

1. How frequent was your child's coughing last night?	honey-Kimia	3.363[a]	.151	3.063	3.663
	Diphenhydramine	3.467[a]	.217	3.035	3.898
	honey_Shahd-eGolha	3.737[a]	.194	3.350	4.124
2. How severe was your child's cough last night?	honey-Kimia	3.694[a]	.126	3.444	3.944
	Diphenhydramine	3.690[a]	.181	3.331	4.050
	honey_Shahd-eGolha	4.242[a]	.162	3.920	4.564
3. How bothersome was last night's cough to your child?	honey-Kimia	3.551[a]	.133	3.287	3.814
	Diphenhydramine	2.891[a]	.191	2.512	3.270
	honey_Shahd-eGolha	3.602[a]	.171	3.263	3.942
4. How much did last night's cough affect your child's ability to sleep?	honey-Kimia	3.107[a]	.175	2.759	3.456
	Diphenhydramine	2.482[a]	.252	1.982	2.983
	honey_Shahd-eGolha	3.434[a]	.226	2.985	3.882
5. How much did last night's cough affect your (parent's) ability to sleep?	honey-Kimia	3.622[a]	.125	3.374	3.869
	Diphenhydramine	2.691[a]	.179	2.335	3.047
	honey_Shahd-eGolha	4.243[a]	.160	3.924	4.562
before_total	honey-Kimia	17.337[a]	.566	16.210	18.463
	Diphenhydramine	15.221[a]	.814	13.602	16.841
	honey_Shahd-eGolha	19.257[a]	.730	17.806	20.709

a. Covariates appearing in the model are evaluated at the following values: age = 3.5287.

Pairwise Comparisons

Dependent Variable	(I) group	(J) group	Mean Difference (I-J)	Std. Error	Sig.[a]	95% Confidence Interval for Difference[a]	
						Lower Bound	Upper Bound
1. How frequent was your child's coughing last night?	honey-Kimia	Diphenhydramine	-.104	.266	.698	-.634	.426
		honey_Shahd-eGolha	-.374	.248	.136	-.868	.120
	Diphenhydramine	honey-Kimia	.104	.266	.698	-.426	.634
		honey_Shahd-eGolha	-.271	.290	.353	-.847	.305
	honey_Shahd-eGolha	honey-Kimia	.374	.248	.136	-.120	.868
		Diphenhydramine	.271	.290	.353	-.305	.847
2. How severe was your child's cough last night?	honey-Kimia	Diphenhydramine	.004	.222	.987	-.437	.444
		honey_Shahd-eGolha	-.548*	.207	.010	-.959	-.137
	Diphenhydramine	honey-Kimia	-.004	.222	.987	-.444	.437
		honey_Shahd-eGolha	-.551*	.241	.025	-1.031	-.072
	honey_Shahd-eGolha	honey-Kimia	.548*	.207	.010	.137	.959

		Diphenhydramine	.551*	.241	.025	.072	1.031
3. How bothersome was last night’s cough to your child?	honey-Kimia	Diphenhydramine	.660*	.234	.006	.195	1.125
		honey_Shahd-eGolha	-.052	.218	.814	-.485	.382
	Diphenhydramine	honey-Kimia	-.660*	.234	.006	-1.125	-.195
		honey_Shahd-eGolha	-.711*	.254	.006	-1.217	-.206
	honey_Shahd-eGolha	honey-Kimia	.052	.218	.814	-.382	.485
		Diphenhydramine	.711*	.254	.006	.206	1.217
4. How much did last night’s cough affect your child’s ability to sleep?	honey-Kimia	Diphenhydramine	.625*	.309	.046	.011	1.240
		honey_Shahd-eGolha	-.326	.288	.261	-.899	.247
	Diphenhydramine	honey-Kimia	-.625*	.309	.046	-1.240	-.011
		honey_Shahd-eGolha	-.951*	.336	.006	-1.619	-.283
	honey_Shahd-eGolha	honey-Kimia	.326	.288	.261	-.247	.899
		Diphenhydramine	.951*	.336	.006	.283	1.619
5. How much did last night’s cough affect your (parent’s) ability to sleep?	honey-Kimia	Diphenhydramine	.931*	.220	.000	.494	1.368
		honey_Shahd-eGolha	-.621*	.205	.003	-1.028	-.213
	Diphenhydramine	honey-Kimia	-.931*	.220	.000	-1.368	-.494
		honey_Shahd-eGolha	-1.552*	.239	.000	-2.027	-1.076
	honey_Shahd-eGolha	honey-Kimia	.621*	.205	.003	.213	1.028
		Diphenhydramine	1.552*	.239	.000	1.076	2.027
before_total	honey-Kimia	Diphenhydramine	2.115*	.999	.037	.128	4.103
		honey_Shahd-eGolha	-1.921*	.932	.042	-3.775	-.067
	Diphenhydramine	honey-Kimia	-2.115*	.999	.037	-4.103	-.128
		honey_Shahd-eGolha	-4.036*	1.087	.000	-6.198	-1.875
	honey_Shahd-eGolha	honey-Kimia	1.921*	.932	.042	.067	3.775
		Diphenhydramine	4.036*	1.087	.000	1.875	6.198

Based on estimated marginal means

a. Adjustment for multiple comparisons: Least Significant Difference (equivalent to no adjustments).

*. The mean difference is significant at the .05 level.

Multivariate Tests

	Value	F	Hypothesis df	Error df	Sig.
Pillai's trace	.556	6.155	10.000	160.000	.000
Wilks' lambda	.510	6.331[a]	10.000	158.000	.000
Hotelling's trace	.834	6.504	10.000	156.000	.000
Roy's largest root	.631	10.089[b]	5.000	80.000	.000

Each F tests the multivariate effect of group. These tests are based on the linearly independent pairwise comparisons among the estimated marginal means.

a. Exact statistic

b. The statistic is an upper bound on F that yields a lower bound on the significance level.

Univariate Tests

Dependent Variable		Sum of Squares	df	Mean Square	F	Sig.
1. How frequent was your child’s coughing last night?	Contrast	2.147	2	1.074	1.152	.321
	Error	77.370	83	.932		
2. How severe was your child’s cough last night?	Contrast	5.271	2	2.636	4.085	.020
	Error	53.558	83	.645		
3. How bothersome was last night’s cough to your child?	Contrast	7.076	2	3.538	4.922	.010
	Error	59.659	83	.719		
4. How much did last night’s cough affect your child’s ability to sleep?	Contrast	10.248	2	5.124	4.085	.020
	Error	104.096	83	1.254		
5. How much did last night’s cough affect your (parent’s) ability to sleep?	Contrast	26.845	2	13.423	21.157	.000
	Error	52.657	83	.634		
before_total	Contrast	181.336	2	90.668	6.908	.002
	Error	1089.344	83	13.125		

The F tests the effect of group. This test is based on the linearly independent pairwise comparisons among the estimated marginal means.

```
USE ALL.
COMPUTE filter_$=(group = 1).
VARIABLE LABEL filter_$ 'group = 1 (FILTER)'.
VALUE LABELS filter_$ 0 'Not Selected' 1 'Selected'.
FORMAT filter_$ (f1.0).
FILTER BY filter_$.
EXECUTE.
T-TEST PAIRS=before_1 before_2 before_3 before_4 before_5 before_total WITH after_1 after_2 after_3 after_4 after_5 after_total (PAI
   RED)
  /CRITERIA=CI(.9500)

  /MISSING=ANALYSIS.
```

T-Test Honey 1

Notes

Output Created	10-Sep-2016 13:54:03

Comments		
Input	Data	F:\DrE,Qods\Dr Ayazi\Honey\Submit\PlosOne\95.5\Resubmit\Honey Data.sav
	Active Dataset	DataSet1
	Filter	group = 1 (FILTER)
	Weight	<none>
	Split File	<none>
	N of Rows in Working Data File	42
Missing Value Handling	Definition of Missing	User defined missing values are treated as missing.
	Cases Used	Statistics for each analysis are based on the cases with no missing or out-of-range data for any variable in the analysis.
Syntax		T-TEST PAIRS=before_1 before_2 before_3 before_4 before_5 before_total WITH after_1 after_2 after_3 after_4 after_5 after_total (PAIRED)<br>/CRITERIA=CI(.9500)<br>/MISSING=ANALYSIS.
Resources	Processor Time	00:00:00.000
	Elapsed Time	00:00:00.000

```
[DataSet1] F:\DrE,Qods\Dr Ayazi\Honey\Submit\PlosOne\95.5\Resubmit\Honey Data.sav
```

Paired Samples Statistics

		Mean	N	Std. Deviation	Std. Error Mean
Pair 1	1. How frequent was your child's coughing last night?	3.38	42	.936	.144
	1. How frequent was your child's coughing last night?	1.95	42	1.188	.183
Pair 2	2. How severe was your child's cough last night?	3.74	42	.828	.128
	2. How severe was your child's cough last night?	2.02	42	1.199	.185
Pair 3	3. How bothersome was last night's cough to your child?	3.60	42	.885	.137

	3. How bothersome was last night's cough to your child?	1.93	42	1.218	.188
Pair 4	4. How much did last night's cough affect your child's ability to sleep?	3.14	42	1.026	.158
	4. How much did last night's cough affect your child's ability to sleep?	.98	42	1.239	.191
Pair 5	5. How much did last night's cough affect your (parent's) ability to sleep?	3.67	42	.816	.126
	5. How much did last night's cough affect your (parent's) ability to sleep?	1.52	42	1.234	.190
Pair 6	before_total	17.5238	42	3.14090	.48465
	after_total	8.4048	42	5.70434	.88020

Paired Samples Correlations

		N	Correlation	Sig.
Pair 1	1. How frequent was your child's coughing last night? & 1. How frequent was your child's coughing last night?	42	.236	.132
Pair 2	2. How severe was your child's cough last night? & 2. How severe was your child's cough last night?	42	.277	.076
Pair 3	3. How bothersome was last night's cough to your child? & 3. How bothersome was last night's cough to your child?	42	.357	.020
Pair 4	4. How much did last night's cough affect your child's ability to sleep? & 4. How much did last night's cough affect your child's ability to sleep?	42	-.055	.730

Pair 5	5. How much did last night's cough affect your (parent's) ability to sleep? & 5. How much did last night's cough affect your (parent's) ability to sleep?	42	.177	.261
Pair 6	before_total & after_total	42	.153	.335

Paired Samples Test

		Paired Differences					t	df	Sig. (2-tailed)
					95% Confidence Interval of the Difference				
		Mean	Std. Deviation	Std. Error Mean	Lower	Upper			
Pair 1	1. How frequent was your child's coughing last night? - 1. How frequent was your child's coughing last night?	1.429	1.328	.205	1.015	1.842	6.973	41	.000
Pair 2	2. How severe was your child's cough last night? - 2. How severe was your child's cough last night?	1.714	1.255	.194	1.323	2.105	8.853	41	.000
Pair 3	3. How bothersome was last night's cough to your child? - 3. How bothersome was last night's cough to your child?	1.667	1.223	.189	1.286	2.048	8.831	41	.000
Pair 4	4. How much did last night's cough affect your child's ability to sleep? - 4. How much did last night's cough affect your child's ability to sleep?	2.167	1.652	.255	1.652	2.681	8.502	41	.000
Pair 5	5. How much did last night's cough affect your (parent's) ability to sleep? - 5. How much did last night's cough affect your (parent's) ability to sleep?	2.143	1.354	.209	1.721	2.565	10.258	41	.000
Pair 6	before_total - after_total	9.11905	6.07756	.93779	7.22515	11.01295	9.724	41	.000

```
USE ALL.
COMPUTE filter_$=(group = 3).
VARIABLE LABEL filter_$ 'group = 3 (FILTER)'.
VALUE LABELS filter_$ 0 'Not Selected' 1 'Selected'.
FORMAT filter_$ (f1.0).
```

```
FILTER BY filter_$.
EXECUTE.
T-TEST PAIRS=before_1 before_2 before_3 before_4 before_5 before_total WITH after_1 after_2 after_3 after_4 after_5 after_total (PAI
   RED)
  /CRITERIA=CI(.9500)

  /MISSING=ANALYSIS.
```

T-Test Honey 2

Notes

Output Created		10-Sep-2016 13:57:14
Comments		
Input	Data	F:\DrE,Qods\Dr Ayazi\Honey\Submit\PlosOne\95.5\Resubmit\Honey Data.sav
	Active Dataset	DataSet1
	Filter	group = 3 (FILTER)
	Weight	<none>
	Split File	<none>
	N of Rows in Working Data File	25
Missing Value Handling	Definition of Missing	User defined missing values are treated as missing.
	Cases Used	Statistics for each analysis are based on the cases with no missing or out-of-range data for any variable in the analysis.
Syntax		T-TEST PAIRS=before_1 before_2 before_3 before_4 before_5 before_total WITH after_1 after_2 after_3 after_4 after_5 after_total (PAIRED)<br>/CRITERIA=CI(.9500)<br>/MISSING=ANALYSIS.
Resources	Processor Time	00:00:00.032
	Elapsed Time	00:00:00.015

```
[DataSet1] F:\DrE,Qods\Dr Ayazi\Honey\Submit\PlosOne\95.5\Resubmit\Honey Data.sav
```

Paired Samples Statistics

		Mean	N	Std. Deviation	Std. Error Mean
Pair 1	1. How frequent was your child’s coughing last night?	3.72	25	.792	.158

	1. How frequent was your child’s coughing last night?	1.84	25	1.546	.309
Pair 2	2. How severe was your child’s cough last night?	4.20	25	.707	.141
	2. How severe was your child’s cough last night?	2.00	25	1.803	.361
Pair 3	3. How bothersome was last night’s cough to your child?	3.56	25	.821	.164
	3. How bothersome was last night’s cough to your child?	1.52	25	1.782	.356
Pair 4	4. How much did last night’s cough affect your child’s ability to sleep?	3.40	25	1.291	.258
	4. How much did last night’s cough affect your child’s ability to sleep?	1.60	25	2.082	.416
Pair 5	5. How much did last night’s cough affect your (parent’s) ability to sleep?	4.20	25	.707	.141
	5. How much did last night’s cough affect your (parent’s) ability to sleep?	1.96	25	1.428	.286
Pair 6	before_total	19.0800	25	3.91493	.78299
	after_total	8.9200	25	8.54849	1.70970

Paired Samples Correlations

		N	Correlation	Sig.
Pair 1	1. How frequent was your child’s coughing last night? & 1. How frequent was your child’s coughing last night?	25	-.311	.131
Pair 2	2. How severe was your child’s cough last night? & 2. How severe was your child’s cough last night?	25	.261	.207
Pair 3	3. How bothersome was last night’s cough to your child? & 3. How bothersome was last night’s cough to your child?	25	-.036	.863

Pair 4	4. How much did last night’s cough affect your child’s ability to sleep? & 4. How much did last night’s cough affect your child’s ability to sleep?	25	.124	.555
Pair 5	5. How much did last night’s cough affect your (parent’s) ability to sleep? & 5. How much did last night’s cough affect your (parent’s) ability to sleep?	25	.297	.149
Pair 6	before_total & after_total	25	.050	.812

Paired Samples Test

		Paired Differences					t	df	Sig. (2-tailed)
					95% Confidence Interval of the Difference				
		Mean	Std. Deviation	Std. Error Mean	Lower	Upper			
Pair 1	1. How frequent was your child’s coughing last night? - 1. How frequent was your child’s coughing last night?	1.880	1.943	.389	1.078	2.682	4.837	24	.000
Pair 2	2. How severe was your child’s cough last night? - 2. How severe was your child’s cough last night?	2.200	1.756	.351	1.475	2.925	6.264	24	.000
Pair 3	3. How bothersome was last night’s cough to your child? - 3. How bothersome was last night’s cough to your child?	2.040	1.989	.398	1.219	2.861	5.128	24	.000
Pair 4	4. How much did last night’s cough affect your child’s ability to sleep? - 4. How much did last night’s cough affect your child’s ability to sleep?	1.800	2.309	.462	.847	2.753	3.897	24	.001
Pair 5	5. How much did last night’s cough affect your (parent’s) ability to sleep? - 5. How much did last night’s cough affect your (parent’s) ability to sleep?	2.240	1.393	.279	1.665	2.815	8.041	24	.000
Pair 6	before_total - after_total	1.01600E1	9.22262	1.84452	6.35309	13.96691	5.508	24	.000

```
USE ALL.
COMPUTE filter_$=(group = 2).
VARIABLE LABEL filter_$ 'group = 2 (FILTER)'.
VALUE LABELS filter_$ 0 'Not Selected' 1 'Selected'.
FORMAT filter_$ (f1.0).
FILTER BY filter_$.
EXECUTE.
T-TEST PAIRS=before_1 before_2 before_3 before_4 before_5 before_total WITH after_1 after_2 after_3 after_4 after_5 after_total (PAI
   RED)
  /CRITERIA=CI(.9500)

  /MISSING=ANALYSIS.
```

T-Test DPH

Notes

Output Created		10-Sep-2016 13:58:15
Comments		
Input	Data	F:\DrE,Qods\Dr Ayazi\Honey\Submit\PlosOne\95.5\Resubmit\Honey Data.sav
	Active Dataset	DataSet1
	Filter	group = 2 (FILTER)
	Weight	<none>
	Split File	<none>
	N of Rows in Working Data File	20
Missing Value Handling	Definition of Missing	User defined missing values are treated as missing.
	Cases Used	Statistics for each analysis are based on the cases with no missing or out-of-range data for any variable in the analysis.
Syntax		T-TEST PAIRS=before_1 before_2 before_3 before_4 before_5 before_total WITH after_1 after_2 after_3 after_4 after_5 after_total (PAIRED) /CRITERIA=CI(.9500) /MISSING=ANALYSIS.
Resources	Processor Time	00:00:00.000
	Elapsed Time	00:00:00.109

```
[DataSet1] F:\DrE,Qods\Dr Ayazi\Honey\Submit\PlosOne\95.5\Resubmit\Honey Data.sav
```

Paired Samples Statistics

		Mean	N	Std. Deviation	Std. Error Mean
Pair 1	1. How frequent was your child's coughing last night?	3.45	20	1.191	.266
	1. How frequent was your child's coughing last night?	2.60	20	1.569	.351
Pair 2	2. How severe was your child's cough last night?	3.65	20	.933	.209
	2. How severe was your child's cough last night?	2.95	20	1.234	.276
Pair 3	3. How bothersome was last night's cough to your child?	2.85	20	.875	.196
	3. How bothersome was last night's cough to your child?	2.20	20	1.056	.236
Pair 4	4. How much did last night's cough affect your child's ability to sleep?	2.45	20	1.099	.246
	4. How much did last night's cough affect your child's ability to sleep?	1.50	20	1.000	.224
Pair 5	5. How much did last night's cough affect your (parent's) ability to sleep?	2.65	20	.933	.209
	5. How much did last night's cough affect your (parent's) ability to sleep?	1.65	20	.933	.209
Pair 6	before_total	15.0500	20	4.43046	.99068
	after_total	10.9000	20	5.34986	1.19627

Paired Samples Correlations

		N	Correlation	Sig.
Pair 1	1. How frequent was your child's coughing last night? & 1. How frequent was your child's coughing last night?	20	.749	.000

Pair 2	2. How severe was your child's cough last night? & 2. How severe was your child's cough last night?	20	.715	.000
Pair 3	3. How bothersome was last night's cough to your child? & 3. How bothersome was last night's cough to your child?	20	.604	.005
Pair 4	4. How much did last night's cough affect your child's ability to sleep? & 4. How much did last night's cough affect your child's ability to sleep?	20	.551	.012
Pair 5	5. How much did last night's cough affect your (parent's) ability to sleep? & 5. How much did last night's cough affect your (parent's) ability to sleep?	20	.396	.084
Pair 6	before_total & after_total	20	.593	.006

Paired Samples Test

		Paired Differences					t	df	Sig. (2-tailed)
					95% Confidence Interval of the Difference				
		Mean	Std. Deviation	Std. Error Mean	Lower	Upper			
Pair 1	1. How frequent was your child's coughing last night? - 1. How frequent was your child's coughing last night?	.850	1.040	.233	.363	1.337	3.655	19	.002
Pair 2	2. How severe was your child's cough last night? - 2. How severe was your child's cough last night?	.700	.865	.193	.295	1.105	3.621	19	.002
Pair 3	3. How bothersome was last night's cough to your child? - 3. How bothersome was last night's cough to your child?	.650	.875	.196	.240	1.060	3.322	19	.004

Pair 4	4. How much did last night's cough affect your child's ability to sleep? - 4. How much did last night's cough affect your child's ability to sleep?	.950	.999	.223	.483	1.417	4.254	19	.000
Pair 5	5. How much did last night's cough affect your (parent's) ability to sleep? - 5. How much did last night's cough affect your (parent's) ability to sleep?	1.000	1.026	.229	.520	1.480	4.359	19	.000
Pair 6	before_total - after_total	4.15000	4.48712	1.00335	2.04997	6.25003	4.136	19	.001

```
FILTER OFF.
USE ALL.
EXECUTE.
GLM Difference_1 Difference_2 Difference_3 Difference_4 Difference_5 Difference_total BY group WITH age
  /METHOD=SSTYPE(3)
  /INTERCEPT=INCLUDE
  /EMMEANS=TABLES(group) WITH(age=MEAN) COMPARE ADJ(LSD)
  /PRINT=DESCRIPTIVE
  /CRITERIA=ALPHA(.05)

  /DESIGN=age group.
```

General Linear Model

Notes

Output Created		10-Sep-2016 13:59:41
Comments		
Input	Data	F:\DrE,Qods\Dr Ayazi\Honey\Submit\PlosOne\95.5\Resubmit\Honey Data.sav
	Active Dataset	DataSet1
	Filter	<none>
	Weight	<none>
	Split File	<none>
	N of Rows in Working Data File	87
Missing Value Handling	Definition of Missing	User-defined missing values are treated as missing.
	Cases Used	Statistics are based on all cases with valid data for all variables in the model.

Syntax		GLM Difference_1 Difference_2 Difference_3 Difference_4 Difference_5 Difference_total BY group WITH age<br>/METHOD=SSTYPE(3)<br>/INTERCEPT=INCLUDE<br>/EMMEANS=TABLES(group) WITH(age=MEAN) COMPARE ADJ(LSD)<br>/PRINT=DESCRIPTIVE<br>/CRITERIA=ALPHA(.05)<br>/DESIGN=age group.
Resources	Processor Time	00:00:00.016
	Elapsed Time	00:00:00.015

```
[DataSet1] F:\DrE,Qods\Dr Ayazi\Honey\Submit\PlosOne\95.5\Resubmit\Honey Data.sav
```

Between-Subjects Factors

		Value Label	N
group	1	honey-Kimia	42
	2	Diphenhydramine	20
	3	honey_Shahd-e Golha	25

Descriptive Statistics

	group	Mean	Std. Deviation	N
Difference_1	honey-Kimia	1.4285714	1.32780503	42
	Diphenhydramine	.8500000	1.03998988	20
	honey_Shahd-eGolha	1.8800000	1.94336478	25
	Total	1.4252874	1.50682312	87
Difference_2	honey-Kimia	1.7142857	1.25495533	42
	Diphenhydramine	.7000000	.86450473	20
	honey_Shahd-eGolha	2.2000000	1.75594229	25
	Total	1.6206897	1.44061824	87
Difference_3	honey-Kimia	1.6666667	1.22308420	42
	Diphenhydramine	.6500000	.87509398	20

	honey_Shahd-eGolha	2.0400000	1.98913717	25
	Total	1.5402299	1.50042318	87
Difference_4	honey-Kimia	2.1666667	1.65155753	42
	Diphenhydramine	.9500000	.99868334	20
	honey_Shahd-eGolha	1.8000000	2.30940108	25
	Total	1.7816092	1.80068002	87
Difference_5	honey-Kimia	2.1428571	1.35379194	42
	Diphenhydramine	1.0000000	1.02597835	20
	honey_Shahd-eGolha	2.2400000	1.39283883	25
	Total	1.9080460	1.37783268	87
Difference_total	honey-Kimia	9.1190476	6.07755720	42
	Diphenhydramine	4.1500000	4.48711606	20
	honey_Shahd-eGolha	10.1600000	9.22261713	25
	Total	8.2758621	7.15068651	87

Multivariate Tests[c]

Effect		Value	F	Hypothesis df	Error df	Sig.
Intercept	Pillai's Trace	.406	10.802[a]	5.000	79.000	.000
	Wilks' Lambda	.594	10.802[a]	5.000	79.000	.000
	Hotelling's Trace	.684	10.802[a]	5.000	79.000	.000
	Roy's Largest Root	.684	10.802[a]	5.000	79.000	.000
age	Pillai's Trace	.086	1.485[a]	5.000	79.000	.204
	Wilks' Lambda	.914	1.485[a]	5.000	79.000	.204
	Hotelling's Trace	.094	1.485[a]	5.000	79.000	.204
	Roy's Largest Root	.094	1.485[a]	5.000	79.000	.204
group	Pillai's Trace	.474	4.964	10.000	160.000	.000
	Wilks' Lambda	.574	5.046[a]	10.000	158.000	.000
	Hotelling's Trace	.657	5.124	10.000	156.000	.000
	Roy's Largest Root	.484	7.745[b]	5.000	80.000	.000

a. Exact statistic

b. The statistic is an upper bound on F that yields a lower bound on the significance level.

c. Design: Intercept + age + group

Tests of Between-Subjects Effects

Source	Dependent Variable	Type III Sum of Squares	df	Mean Square	F	Sig.
Corrected Model	Difference_1	14.305[a]	3	4.768	2.187	.096
	Difference_2	25.715[b]	3	8.572	4.657	.005
	Difference_3	23.120[c]	3	7.707	3.752	.014
	Difference_4	20.615[d]	3	6.872	2.209	.093
	Difference_5	21.823[e]	3	7.274	4.269	.007
	Difference_total	470.428[f]	3	156.809	3.314	.024
Intercept	Difference_1	14.605	1	14.605	6.699	.011
	Difference_2	34.669	1	34.669	18.836	.000
	Difference_3	24.687	1	24.687	12.018	.001
	Difference_4	30.753	1	30.753	9.884	.002
	Difference_5	40.213	1	40.213	23.598	.000
	Difference_total	705.712	1	705.712	14.916	.000
age	Difference_1	2.516	1	2.516	1.154	.286
	Difference_2	.004	1	.004	.002	.964
	Difference_3	.354	1	.354	.172	.679
	Difference_4	.547	1	.547	.176	.676
	Difference_5	.262	1	.262	.153	.696
	Difference_total	11.364	1	11.364	.240	.625
group	Difference_1	11.919	2	5.960	2.733	.071
	Difference_2	25.703	2	12.851	6.982	.002
	Difference_3	22.450	2	11.225	5.465	.006
	Difference_4	18.432	2	9.216	2.962	.057
	Difference_5	20.915	2	10.457	6.137	.003
	Difference_total	446.365	2	223.183	4.717	.011
Error	Difference_1	180.960	83	2.180		
	Difference_2	152.768	83	1.841		
	Difference_3	170.489	83	2.054		
	Difference_4	258.236	83	3.111		
	Difference_5	141.441	83	1.704		
	Difference_total	3926.951	83	47.313		
Total	Difference_1	372.000	87			
	Difference_2	407.000	87			

	Difference_3	400.000	87			
	Difference_4	555.000	87			
	Difference_5	480.000	87			
	Difference_total	10356.000	87			
Corrected Total	Difference_1	195.264	86			
	Difference_2	178.483	86			
	Difference_3	193.609	86			
	Difference_4	278.851	86			
	Difference_5	163.264	86			
	Difference_total	4397.379	86			

a. R Squared = .073 (Adjusted R Squared = .040)

b. R Squared = .144 (Adjusted R Squared = .113)

c. R Squared = .119 (Adjusted R Squared = .088)

d. R Squared = .074 (Adjusted R Squared = .040)

e. R Squared = .134 (Adjusted R Squared = .102)

f. R Squared = .107 (Adjusted R Squared = .075)

Estimated Marginal Means

group

Estimates

				95% Confidence Interval	
Dependent Variable	group	Mean	Std. Error	Lower Bound	Upper Bound
Difference_1	honey-Kimia	1.389[a]	.231	.930	1.848
	Diphenhydramine	.886[a]	.332	.226	1.546
	honey_Shahd-eGolha	1.918[a]	.297	1.326	2.509
Difference_2	honey-Kimia	1.716[a]	.212	1.294	2.138
	Diphenhydramine	.699[a]	.305	.092	1.305
	honey_Shahd-eGolha	2.199[a]	.273	1.655	2.742
Difference_3	honey-Kimia	1.652[a]	.224	1.206	2.097
	Diphenhydramine	.664[a]	.322	.023	1.304
	honey_Shahd-eGolha	2.054[a]	.289	1.480	2.628
Difference_4	honey-Kimia	2.148[a]	.276	1.600	2.697
	Diphenhydramine	.967[a]	.396	.178	1.756

	honey_Shahd-eGolha	1.818[a]	.355	1.111	2.524
Difference_5	honey-Kimia	2.130[a]	.204	1.724	2.536
	Diphenhydramine	1.012[a]	.293	.428	1.595
	honey_Shahd-eGolha	2.252[a]	.263	1.729	2.775
Difference_total	honey-Kimia	9.035[a]	1.075	6.896	11.173
	Diphenhydramine	4.227[a]	1.546	1.152	7.302
	honey_Shahd-eGolha	10.240[a]	1.385	7.485	12.995

a. Covariates appearing in the model are evaluated at the following values: age = 3.5287.

Pairwise Comparisons

Dependent Variable	(I) group	(J) group	Mean Difference (I-J)	Std. Error	Sig.[a]	95% Confidence Interval for Difference[a] Lower Bound	Upper Bound
Difference_1	honey-Kimia	Diphenhydramine	.503	.407	.221	-.308	1.313
		honey_Shahd-eGolha	-.529	.380	.168	-1.284	.227
	Diphenhydramine	honey-Kimia	-.503	.407	.221	-1.313	.308
		honey_Shahd-eGolha	-1.031*	.443	.022	-1.912	-.150
	honey_Shahd-eGolha	honey-Kimia	.529	.380	.168	-.227	1.284
		Diphenhydramine	1.031*	.443	.022	.150	1.912
Difference_2	honey-Kimia	Diphenhydramine	1.017*	.374	.008	.273	1.762
		honey_Shahd-eGolha	-.483	.349	.170	-1.177	.211
	Diphenhydramine	honey-Kimia	-1.017*	.374	.008	-1.762	-.273
		honey_Shahd-eGolha	-1.500*	.407	.000	-2.309	-.690
	honey_Shahd-eGolha	honey-Kimia	.483	.349	.170	-.211	1.177
		Diphenhydramine	1.500*	.407	.000	.690	2.309
Difference_3	honey-Kimia	Diphenhydramine	.988*	.395	.014	.202	1.775
		honey_Shahd-eGolha	-.402	.369	.278	-1.136	.331
	Diphenhydramine	honey-Kimia	-.988*	.395	.014	-1.775	-.202
		honey_Shahd-eGolha	-1.390*	.430	.002	-2.246	-.535
	honey_Shahd-eGolha	honey-Kimia	.402	.369	.278	-.331	1.136
		Diphenhydramine	1.390*	.430	.002	.535	2.246
Difference_4	honey-Kimia	Diphenhydramine	1.181*	.487	.017	.213	2.149
		honey_Shahd-eGolha	.331	.454	.468	-.572	1.233
	Diphenhydramine	honey-Kimia	-1.181*	.487	.017	-2.149	-.213
		honey_Shahd-eGolha	-.851	.529	.112	-1.903	.202

	honey_Shahd-eGolha	honey-Kimia	-.331	.454	.468	-1.233	.572
		Diphenhydramine	.851	.529	.112	-.202	1.903
Difference_5	honey-Kimia	Diphenhydramine	1.118*	.360	.003	.402	1.835
		honey_Shahd-eGolha	-.122	.336	.717	-.790	.546
	Diphenhydramine	honey-Kimia	-1.118*	.360	.003	-1.835	-.402
		honey_Shahd-eGolha	-1.240*	.392	.002	-2.019	-.461
	honey_Shahd-eGolha	honey-Kimia	.122	.336	.717	-.546	.790
		Diphenhydramine	1.240*	.392	.002	.461	2.019
Difference_total	honey-Kimia	Diphenhydramine	4.808*	1.898	.013	1.033	8.582
		honey_Shahd-eGolha	-1.205	1.770	.498	-4.725	2.314
	Diphenhydramine	honey-Kimia	-4.808*	1.898	.013	-8.582	-1.033
		honey_Shahd-eGolha	-6.013*	2.064	.005	-10.117	-1.909
	honey_Shahd-eGolha	honey-Kimia	1.205	1.770	.498	-2.314	4.725
		Diphenhydramine	6.013*	2.064	.005	1.909	10.117

Based on estimated marginal means

a. Adjustment for multiple comparisons: Least Significant Difference (equivalent to no adjustments).

*. The mean difference is significant at the .05 level.

Multivariate Tests

	Value	F	Hypothesis df	Error df	Sig.
Pillai's trace	.474	4.964	10.000	160.000	.000
Wilks' lambda	.574	5.046[a]	10.000	158.000	.000
Hotelling's trace	.657	5.124	10.000	156.000	.000
Roy's largest root	.484	7.745[b]	5.000	80.000	.000

Each F tests the multivariate effect of group. These tests are based on the linearly independent pairwise comparisons among the estimated marginal means.

a. Exact statistic

b. The statistic is an upper bound on F that yields a lower bound on the significance level.

Univariate Tests

Dependent Variable		Sum of Squares	df	Mean Square	F	Sig.
Difference_1	Contrast	11.919	2	5.960	2.733	.071
	Error	180.960	83	2.180		
Difference_2	Contrast	25.703	2	12.851	6.982	.002

	Error	152.768	83	1.841		
Difference_3	Contrast	22.450	2	11.225	5.465	.006
	Error	170.489	83	2.054		
Difference_4	Contrast	18.432	2	9.216	2.962	.057
	Error	258.236	83	3.111		
Difference_5	Contrast	20.915	2	10.457	6.137	.003
	Error	141.441	83	1.704		
Difference_total	Contrast	446.365	2	223.183	4.717	.011
	Error	3926.951	83	47.313		

The F tests the effect of group. This test is based on the linearly independent pairwise comparisons among the estimated marginal means.
